# Neurally adjusted ventilatory assist in patients with acute respiratory failure: study protocol for a randomized controlled trial

**DOI:** 10.1186/s13063-016-1625-5

**Published:** 2016-10-13

**Authors:** Jesús Villar, Javier Belda, Jesús Blanco, Fernando Suarez-Sipmann, José Manuel Añón, Lina Pérez-Méndez, Carlos Ferrando, Dácil Parrilla, Raquel Montiel, Ruth Corpas, Elena González-Higueras, David Pestaña, Domingo Martínez, Lorena Fernández, Marina Soro, Miguel Angel García-Bello, Rosa Lidia Fernández, Robert M. Kacmarek, the NAVa In Acute respiraTORy failure (NAVIATOR) Network

**Affiliations:** 1 CIBER de Enfermedades Respiratorias, Instituto de Salud Carlos III, Monforte de Lemos 3-5, Pabellon 11, 28029 Madrid, Spain; 2 Multidisciplinary Organ Dysfunction Evaluation Research Network, Research Unit, Hospital Universitario Dr. Negrín, Barranco de la Ballena s/n, 4th Floor-South Wing, 35019 Las Palmas de Gran Canaria, Spain; 3 Keenan Research Center for Biomedical Science at the Li Ka Shing Knowledge Institute, St. Michael’s Hospital, 30 Bond St, Toronto, ON M5B 1W8 Canada; 4 Department of Anesthesiology, Hospital Clínico Universitario de Valencia, Avda. Blasco Ibañez 17, 46010 Valencia, Spain; 5 Intensive Care Unit, Hospital Universitario Río Hortega, Calle Dulzaina, 2, 47012 Valladolid, Spain; 6 Hedenstierna Laboratory, Department of Surgical Sciences, Uppsala University Hospital, Akademiska Sjukhuset, Ing 40, Tr 3, SE-75185 Uppsala, Sweden; 7 Intensive Care Unit, Hospital Virgen de La Luz, Hermandad de Donantes de Sangre s/n, 16002 Cuenca, Spain; 8 Division of Clinical Epidemiology and Biostatistics, Research Unit, Hospital Universitario NS de Candelaria, Carretera General del Rosario 145, 38010 Santa Cruz de Tenerife, Spain; 9 Intensive Care Unit, Hospital Universitario NS de Candelaria, Carretera General del Rosario 145, 38010 Santa Cruz de Tenerife, Spain; 10 Intensive Care Unit, Hospital General NS del Prado, Carretera de Madrid, Km. 114, 45600 Talavera de la Reina, Toledo Spain; 11 Department of Anesthesiology, Hospital Universitario Ramón y Cajal, Carretera de Colmenar Viejo, Km. 9,100, 28034 Madrid, Spain; 12 Intensive Care Unit, Hospital Universitario Virgen de la Arrixaca, Carretera Madrid-Cartagena s/n, 30120 El Palmar, Murcia Spain; 13 Division of Biostatistics, Research Unit, Hospital Universitario Dr. Negrín, Barranco de la Ballena s/n, 35019 Las Palmas de Gran Canaria, Spain; 14 Department of Respiratory Care, Massachusetts General Hospital, 55 Fruit St, Boston, MA 02114 USA; 15 Department of Anesthesiology, Harvard University, 55 Fruit Street Gray-Bigelow 444, Boston, MA 02144 USA

**Keywords:** Acute respiratory failure, Neurally adjusted ventilatory assist, Ventilator-free days, Lung-protective ventilation, Assist ventilation, Liberation from mechanical ventilation

## Abstract

**Background:**

Patient-ventilator asynchrony is a common problem in mechanically ventilated patients with acute respiratory failure. It is assumed that asynchronies worsen lung function and prolong the duration of mechanical ventilation (MV). Neurally Adjusted Ventilatory Assist (NAVA) is a novel approach to MV based on neural respiratory center output that is able to trigger, cycle, and regulate the ventilatory cycle. We hypothesized that the use of NAVA compared to conventional lung-protective MV will result in a reduction of the duration of MV. It is further hypothesized that NAVA compared to conventional lung-protective MV will result in a decrease in the length of ICU and hospital stay, and mortality.

**Methods/design:**

This is a prospective, multicenter, randomized controlled trial in 306 mechanically ventilated patients with acute respiratory failure from several etiologies. Only patients ventilated for less than 5 days, and who are expected to require prolonged MV for an additional 72 h or more and are able to breathe spontaneously, will be considered for enrollment. Eligible patients will be randomly allocated to two ventilatory arms: (1) conventional lung-protective MV (*n* = 153) and conventional lung-protective MV with NAVA (*n* = 153). Primary outcome is the number of ventilator-free days, defined as days alive and free from MV at day 28 after endotracheal intubation. Secondary outcomes are total length of MV, and ICU and hospital mortality.

**Discussion:**

This is the first randomized clinical trial examining, on a multicenter scale, the beneficial effects of NAVA in reducing the dependency on MV of patients with acute respiratory failure.

**Trial registration:**

ClinicalTrials.gov website (NCT01730794). Registered on 15 November 2012.

**Electronic supplementary material:**

The online version of this article (doi:10.1186/s13063-016-1625-5) contains supplementary material, which is available to authorized users.

## Background

The act of taking a breath is controlled by the respiratory center of the brain, which decides the characteristics of each breath, its timing, and its size. The respiratory center sends a signal along the phrenic nerve that excites the diaphragmatic muscle cells, leading to muscle contraction and descent of the diaphragmatic dome. As a result, the pressure in the airway drops causing an inflow of air into the lungs. Patient-ventilator asynchrony is a common problem in mechanically ventilated patients with acute respiratory failure (ARF). Asynchrony has been frequently documented in both volume and pressure assist/control (A/C) as well as pressure support ventilation. Two recent clinical studies have reported an increased length of mechanical ventilation (MV) requirement in patients with an Asynchrony Index (AI) ≥ 10 % versus those with an AI < 10 %. AI is defined as the ratio between number of asynchronies per minute/total respiratory rate × 100. In one study, patients with an AI ≥ 10 % required 25.5 days of ventilatory support versus 7.5 days in those with an AI < 10 % [1], while in the other study the number of ventilator-free days (VFDs) was 21 days (AI ≥ 10 %) versus 25 (AI < 10 %) [2]. In a more recent study in 50 patients all triggering the ventilator, every patient had multiple periods during the day where the AI was over 5 % and some had an AI as high as 40 % [3]. Of even greater concern is the fact the clinicians have a very difficult time identifying the presence of asynchrony and determining the AI at the bedside [4].

A new mode of ventilation, Neurally Adjusted Ventilatory Assist (NAVA) has been recently introduced [5–16]. With NAVA, the electrical activity of the diaphragm (Edi) is captured, fed to the ventilator and used to assist the patient’s breathing in synchrony with, and in proportion to, the patient’s own efforts regardless of patient category or size. NAVA triggers, cycles and regulates gas delivery based on the diaphragmatic electromyography (EMG) signal via a specially designed nasogastric tube (Edi). As the work of the ventilator and the diaphragm is controlled by the same signal, coupling between the diaphragm and the ventilator is synchronized simultaneously. As a result, synchrony should be markedly improved with NAVA since neither air leaks nor auto-positive end-expiratory pressure (auto-PEEP) should affect the ability of the ventilator to trigger, deliver gas or cycle.

We hypothesized that the use of NAVA will improve patient-ventilator interaction, will make the transition to spontaneous breathing much quicker and easier, and will result in an increase in VFDs in patients with ARF when compared to conventional lung-protective MV. If our hypothesis is correct, the use of NAVA will decrease the duration of MV, the length of intensive care unit (ICU) stay, ventilator-associated complications, and has the potential to increase overall survival of patients with ARF.

## Methods/design

### Justification of the study

Patient-ventilator asynchrony is a common problem in mechanically ventilated patients with ARF who are receiving assisted ventilation. Based on new data, all patients managed with conventional modes of MV have an AI > 5 % at various points during the day [3]. NAVA is a unique approach to MV based on neural respiratory output, providing a smooth transition to natural breathing. NAVA better assures that the patient’s breathing is in synchrony with, and in proportion to, the patient’s own efforts, regardless of patient category or size.

We justify the need for our study based on the fact that NAVA can provide a smooth transition to natural breathing. We hypothesized that the use of NAVA compared to conventional lung-protective MV will result in a decrease in the number of days of MV. It is further hypothesized that NAVA will result in a decrease in the length of ICU and hospital stay. The goal of this study is to compare the ability of NAVA versus conventional lung-protective MV to provide invasive ventilatory support during ARF in adults who are expected to require ventilatory support for at least 72 h.

### Study design

This study is a prospective, multicenter, randomized controlled, clinical trial in 306 adult patients (male and female) with ARF admitted to a network of 18 ICUs from university and community hospitals in Spain (Appendix 1).

The trial has been designed in accordance with the fundamental principles established in the Declaration of Helsinki, the Convention of the European Council related to human rights and biomedicine, and the Universal Declaration of UNESCO on the human genome and human rights, and within the requirements established by the Spanish legislation (Law 14/2007, Law 15/1999) in the field of biomedical research, the protection of personal data, and bioethics. The study was registered on 15 November 2012 at https://clinicaltrials.gov/ct2/show/NCT01730794 with the identification number NCT01730794. The study was approved by a referral Ethics Committee (Hospital Clínico Universitario de Valencia, Valencia, Spain) and the Institutional Review Boards (IRBs) of all participating hospitals (Additional file 1). For inclusion into the study, signed written informed consent from the patient or the patient’s personal legal representative will be obtained (Additional file 2). See Additional file 3 for the Standard Protocol Items: Recommendations for Interventional Trials (SPIRIT) checklist of the study protocol.

### Study population

To be eligible for inclusion into this study (day 0), each patient must fulfill the following inclusion criteria during screening and prior to enrollment into the trial: be aged 18 years or older, have hypoxemic or hypercapnic ARF, be intubated and mechanically ventilated for less than 5 days but expected to be ventilated for at least 72 h, and be able to spontaneously trigger the ventilator. Patients will be excluded from study participation if any of the following criteria are present: moderate or severe acute respiratory distress syndrome (ARDS) [17], the presence of three or more organ system failures, require noninvasive ventilation, be unable to spontaneously breathe, or have a poor short-term prognosis (defined as a high risk of death in the next 3 months), neuromuscular or neurological disease, lack of informed consent, and any medical/surgical contraindication.

### Enrollment into the study and randomization

Although the calculation of AI is not mandatory for patient enrollment after patient consent, it is recommended to perform (and save) a 1- to 15-min tracing recording of the patients’ pressure and flow waveforms during the mode of MV that they were receiving at the time of randomization. At the end of the study, in a randomly selected 10 % of enrolled patients, the AI will be calculated based on the following: (1) number of missed triggers (an airway pressure decrease of at least 0.5 cmH_2_O that does not result in triggering of the ventilator), (2) number of double triggers (two consecutive breaths without an exhalation between them), (3) auto-triggers (ventilator triggering to inspiration without a decrease in airway pressure of at least 0.5 cmH_2_O), (4) short inspiratory time (inspiratory time less than half of the mean inspiratory time), and (5) long inspiratory time (inspiratory time more than twice the mean inspiratory time). We will determine the AI using the formula from Thille et al. [1]:

$$ \mathrm{AI}\ \left(\%\right) = \mathrm{number}\ \mathrm{of}\ \mathrm{asynchrony}\ \mathrm{events}/\mathrm{total}\ \mathrm{respiratory}\ \mathrm{rate}\ \left(\mathrm{which}\ \mathrm{includes}\ \mathrm{ventilatory}\ \mathrm{cycles} + \mathrm{wasted}\ \mathrm{efforts}\right) \times 100. $$

Thille et al. placed a pressure transducer and pneumotachograph at the airway and recorded breaths for 30 min. This is impractical to do as an entry criterion for a multicenter randomized controlled trial. However, all possible efforts will be made to record representative waveform tracings using the Servo-tracker software, or the Servo-i ventilator PCMCIA recording cards, or by any other means during a 1- to 15-min period of the ventilatory tracing before enrollment.

**Fig. 1:**
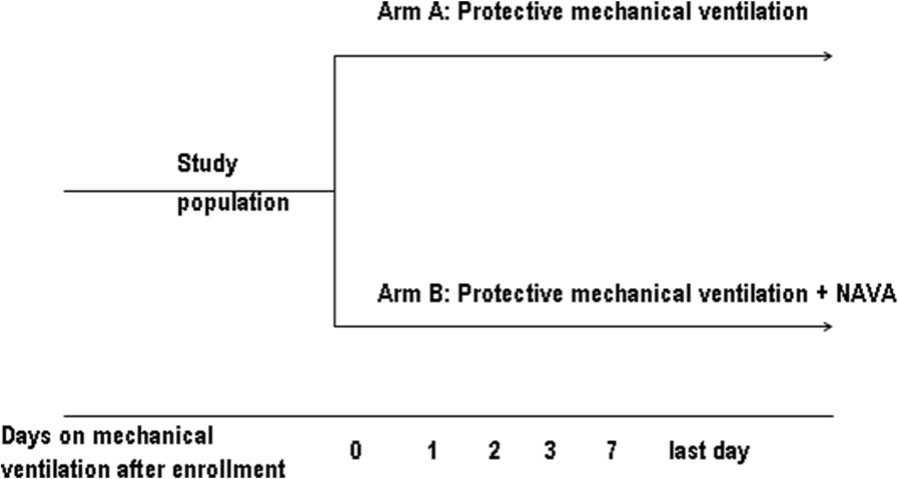
Study design diagram

Eligible and informed consented patients who are expected to be ventilated for at least 72 h will be enrolled and randomly allocated to two arms: (1) NAVA or (2) conventional lung-protective MV, within the first 5 days (120 h) after endotracheal intubation (Fig. 1).

The randomization list was done by the statistician of the study (MAGB), according to a computer-generated random number table based on a 1:1 allocation. This list was generated when the investigators and all Ethics Committees and IRBs of the participating centers had approved the study design. Randomization is stratified by center to ensure an equal distribution of patients in each arm by each ICU. Patients are randomly allocated to the two arms of the study (NAVA or no NAVA) by the clinical investigator in each participating ICU according to the precise written allocation contained inside a prenumbered, opaque, sealed envelope sent in blocks of ten envelopes to each participating ICU. Investigators must respect the numerical sequence of the envelopes. Only two members of the Trial Management Team have access to the randomization list: the project manager and the statistician. They keep the randomization list in their private office at the Research Unit of the Coordinating Center. The coordinating center does not enroll patients.

Our study characteristics do not allow the blinding of investigators to the intervention being tested. Once a participating ICU randomizes a patient, their investigators must contact the project manager of the study to inform and confirm the randomization number, the correct sequence of the prenumbered envelope, and the intervention arm. Screening, enrollment, randomization, treatment initiation, and follow-up of randomized patients is performed by the investigators and attending physicians in each participating ICU. Subsequent blocks of ten envelopes are sent to those participating ICUs with high enrollment rates. Participating clinicians do not know whether the pattern of stratification by center is for every 10, 20, or more envelopes during the study period.

### Ventilatory management

In the conventional lung-protective MV group, patients will be ventilated using either volume assist/control (A/C), pressure A/C, pressure support (PS), pressure-regulated volume control (PRVC), or volume support (VS) at the discretion of the medical team with tidal volumes (VT) in the 4 to 8 ml/kg predicted body weight (PBW) range and plateau pressure or pressure (control or support) level setting of 30 cmH_2_O or less. During A/C the backup rate must be set to insure that more than 90 % of breaths are triggered by the patient. In the weaning phase of ventilatory support, a VT of up to 10 ml/kg will be acceptable. Volume and pressure ventilation should be optimized by careful adjustment of peak flow and inspiratory time (volume ventilation), and rise time and termination criteria (pressure ventilation) to insure maximum patient-ventilator synchrony. In volume ventilation, inspiratory time should be less than 1.0 s and peak flow should be high enough to avoid any concavity during the initial part of inspiration. In pressure ventilation, rise time should also be set to avoid any concavity during the initial part of inspiration, and the patient’s neural inspiratory time and the ventilator’s inspiratory time should end simultaneously by careful setting of inspiratory time or termination criteria. Trigger sensitivity should be set to insure the minimal effort that does not result in auto-triggering.

For the purpose of this study, patients allocated to the NAVA group will be ventilated with Servo-i ventilators (Maquet-Getinge, Solna, Sweden). One NAVA catheter size will be used for this study: Edi catheter 16 Fr (Maquet-Getinge, Solna, Sweden). For details on guidelines for positioning the Edi Catheter, initial NAVA settings, and subsequent adjustments of NAVA, see Additional file 4. The NAVA level will be set initially at zero, then the maximum Edi will be determined as the average level over the next at least three to five breaths without ventilatory support but with 5 cmH_2_O of PEEP. The actual NAVA level will then be titrated by the clinician to achieve the following: (1) an Edi equal to approximately 50 % of the maximum Edi, (2) an average VT of between 4 to 8 ml/kg PBW, and (3) an average respiratory rate of between 15 and 40 per min. In addition, the trigger sensitivity should be set as sensitive as possible without causing auto-triggering and the maximum pressure limit in NAVA should be set at 40 cmH_2_O. The NAVA catheter should be changed every 5 days.

In both groups, PEEP will be set in hypoxemic ventilatory failure patients at a minimum of 5 cmH_2_O. Since none of these patients will have moderate or severe ARDS, it is expected that PEEP levels in most patients will be set between 5 and 15 cmH_2_O based on the clinical judgment of the attending physician. The primary exception to this is patients with marked obesity who may require PEEP levels of up to 15 to 20 cmH_2_O. However, a high-PEEP-low-FiO_2_ approach is expected with all of these patients. In chronic obstructive pulmonary disease (COPD) patients, PEEP will be set to offset auto-PEEP. That is, PEEP will be increased to the level that insures that the vast majority of patient efforts result in triggering of the ventilator. In congestive heart failure patients, PEEP will be set at 8 to 12 cmH_2_O based on the patient’s hemodynamic status.

In both arms, FiO_2_ will be set to insure a PaO_2_ of 60 to 80 mmHg after the setting of PEEP. In patients who leave the ICU for any reason, the randomized approach should be continued during their travel. Patients who require sedation or anesthesia for procedures should be placed back on the appropriately randomized mode as soon as they are able to breathe spontaneously. Throughout this study, the applied VT will be based on the patients’ PBW. The following formulas are to be used to calculate PBW (kg):

$$ \mathrm{P}\mathrm{B}\mathrm{W} = 50.0 + \left[0.91 \times \left(\mathrm{height}\ \mathrm{in}\ \mathrm{cm} - 152\right)\right]\ \mathrm{f}\mathrm{o}\mathrm{r}\ \mathrm{men}, $$

and

$$ \mathrm{P}\mathrm{B}\mathrm{W} = 45.5 + \left[0.91 \times \left(\mathrm{height}\ \mathrm{in}\ \mathrm{cm} - 152\right)\right]\ \mathrm{f}\mathrm{o}\mathrm{r}\ \mathrm{women}. $$

In all patients, weaning will be performed by a spontaneous breathing trial (SBT) [18]. Following extubation in both groups, noninvasive ventilation will be applied for 24 to 48 h if patients are over 65 years old, have COPD or congestive heart failure, have an ineffective cough and excessive secretions, have had at least one weaning failure, more than one comorbid condition, upper airway obstruction, or an APACHE II score > 12 on the day of extubation. In the control arm noninvasive PS will be applied, and in the NAVA arm noninvasive NAVA will be applied.

If, after 2 h post extubation, an FiO_2_ > 0.40 is required, patients will be maintained on continuous positive airway pressure (CPAP) at 10 cmH_2_O or bilevel positive airway pressure (BiPAP) at clinically determined settings with peak inspiratory pressure of 20 cmH_2_O or less via face mask until they are able to maintain a PaO_2_ > 60 mmHg on an FiO_2_ ≤ 0.40. If patients do not respond to CPAP/BiPAP within 2 h, reintubation for those considered failing should not be delayed. Patients in the NAVA group should not have the NAVA catheter removed until BiPAP or CPAP has been discontinued. If these patients are reintubated they are to return to NAVA.

### Daily spontaneous breathing trial (SBT) assessment/performance for both groups

Prior to the start of a SBT the patient should demonstrate: (1) a partial reversal of the underlying cause of ARF, (2) SpO_2_ ≥ 88 % or PaO_2_ ≥ 55 mmHg with FiO_2_ ≤ 0.40 and PEEP of 8 cmH_2_O or less, (3) hemodynamic stability, (4) a level of sedation appropriate for SBT, and (5) the ability to spontaneously breathe.

The SBT will be conducted for 30 to 60 min. The SBT should be conducted while attached to the ventilator with the ventilator set at zero or 5 cmH_2_O pressure support and zero or 5 cmH_2_O of CPAP or via a T-piece with the same FiO_2_ as during ventilation. Any of the following criteria identify failure of the SBT: (1) respiratory rate (RR) ≥ 35/min for 5 min or longer, (2) sustained SpO_2_ < 88 %, (3) mean arterial pressure sustained < 60 or > 120 mmHg, (4) ischemic changes on the electrocardiogram, (5) new onset of paradoxical breathing, accessory muscle use, nasal flaring, etc., and (6) agitation, diaphoresis, anxiety that does not resolve with reassurance. Those patients successfully completing a 30- to 60-min SBT will be extubated unless there is a specific reason not to extubate. The reason for not extubating a patient after a successful SBT will be documented.

### General care and procedures for both groups of patients

All participating patients, regardless of the study arm into which they are randomized, will be monitored and managed following general standard of care practices aimed at maintaining optimal conditions. Measurement of auto-PEEP (the static end-expiratory pressure following an end-expiratory pause of at least 2.0 s) and measurement of plateau pressure (the static end-inspiratory pressure following an end-inspiratory pause of at least 2.0 s) will be performed daily as needed. Active humidification or heat and moisture exchangers (HMEs) may be used on patients on either group. Mechanical dead space will be minimized on all patients. Use of inline suction catheters is up to the discretion of the investigator; however, they are recommended in all patients. Normal body temperature will be maintained. Ventilator circuits and inline suction catheters do not need to be changed on a regular basis. During airway suctioning the following will be avoided: instillation of saline, manual ventilation, and aggressive suction. Suctioning will only occur when secretions are present; routine suctioning should be avoided. Ideally metered-dose inhalers (MDIs) or Aerogen nebulizers should be used for aerosol therapy. A spacer should be maintained in the inspiratory limb. If a small volume nebulizer is used, a one-way valve T-piece will be used to avoid disconnection of the circuit. Correction for compressible volume of the circuit will be activated in all patients regardless of arm of the study.

Minimal sedation and appropriate analgesia will be maintained in all patients. The sedative/analgesic regimen and dose will be those selected by the managing physician at all times according to the needs of the patient and unit policies. Sedation should be titrated according to any of the commonly used sedation scales: the Riker Sedation-Agitation Scale (SAS) [19], the Ramsay Sedation Scale [20], or the Richmond Agitation Scale (RASS) score [21] (see Additional file 4). Appropriate analgesia will be given so that no more than minimal sedation will be necessary. The specific drugs used and dosing regimen will be left up to the individual investigator. Drugs used and dose per kg will be recorded on the data forms.

When an appropriate fluid challenge fails to restore adequate blood pressure and organ perfusion, therapy with vasopressor agents should be started. Either norepinephrine or dopamine (through a central catheter as soon as available) is the first-choice vasopressor agent to correct hypotension in septic shock. Vasopressin may be considered in patients with refractory shock despite adequate fluid resuscitation and high-dose conventional vasopressors. These recommendations may be revised as the new surviving sepsis guidelines become available. In patients with low cardiac output despite adequate fluid resuscitation, dobutamine may be used to increase cardiac output. If used in the presence of low blood pressure it should be combined with vasopressor therapy. A strategy of increasing Cardiac Index to achieve an arbitrarily predefined elevated level is not recommended.

Enteral nutrition should be provided as soon as it is deemed safe by the treating physicians. Exogenous insulin should be provided with the goal of achieving a blood glucose level below 150 mg/dl for the first 3 days of critical illness. If, after 3 days, enteral nutrition has been established and other resuscitation measures have been provided, a goal of normoglycemia (110–140 mg/dl) should be considered. This approach should minimize the potential impact of hypoglycemia. Ongoing randomized controlled trials may change these recommendations, in which case we will provide protocol amendments if necessary.

Data on lung mechanics, gas exchange, and hemodynamics will be gathered before applying study settings, then after applying study settings at times 0, 4 h, 24 h, 48 h, 96 h, 7 days, 10 days, 14 days, and every 7 days thereafter and on the last day of invasive ventilatory support (Table 1). At initiation of the protocol and at 4 h, data collected will represent one point in time, that is individual data will be coupled. However, each day after that data will not be coupled since the highest and lowest value for each variable will be recorded. It is expected that this data will be collected between 8 a.m. and 12 noon each day. Also, at this time the maximum Edi (the peak inspiratory value) will be reassessed and readjustments of MV will be made if necessary. Patients will be followed-up (alive or dead) until ICU discharge. In addition, we will record hospital length of stay and mortality.

**Table 1:** Schedule of events

Events	Screening Day	Randomization (Day 0)	Day 1	Day 2	Day 3	Day 7	Day 10	Last day on MV
Study procedures								
Informed consent form	X							
Medical history	X	X	X	X	X	X	X	X
Demographics	X							
Intervention								
Treatment/intervention		X	X	X	X	X	X	X
Blood chemistry								
Blood gases	X	X	X	X	X	X	X	X
Efficacy measure								
Lung mechanics	X	X	X	X	X	X	X	X
NAVA levels	X	X	X	X	X	X	X	X
Hemodynamic data	X	X	X	X	X	X	X	X
Radiological tests								
Chest X-ray	X	X	X	X	X	X	X	X

*MV* mechanical ventilation, *NAVA* Neurally Adjusted Ventilatory Assist

### Primary and secondary outcomes

The primary outcome of interest is the number of VFDs, defined as days alive and free from MV at day 28 from the onset of MV and from the day of randomization. For subjects ventilated for 28 days or more and for subjects who die, VFDs is 0. The secondary outcomes of interest include: length of invasive MV, length of post-extubation noninvasive ventilation, total assisted VFDs (invasive plus noninvasive), length of ICU and hospital stay, and ICU and hospital mortality, development of extrapulmonary organ failure (any organ failure developed during the study that was not present at the time of enrollment into the study) [22], prevalence of barotrauma (defined as the presence of any extrapulmonary air that was not present at study enrollment), ventilator-associated pneumonia (development of a pneumonia 48 h after study entry), development of ARDS after enrollment into the study, as defined by the American-European Consensus Conference (AECC) criteria for ARDS [23] or as moderate/severe ARDS by the Berlin criteria [17], and time from first SBT to extubation.

### Sample size calculations and interim analysis

It is anticipated that the average patient with asynchrony who is enrolled into this trial will have 21 VFDs with a standard deviation (SD) of 6 VFDs, based on data from Wit et al. [2]. Considering the impact of improved synchrony on length of MV, we anticipate that NAVA will increase VFDs by 2 days. No patient loss has been considered. We will only analyze patients who are enrolled and randomized. The power analysis has been performed according to Schoenfeld et al. [24]. Thus, the sample size with an alpha of 0.05 and a beta of 0.2 (80 % power) is 153 patients in each group or a total of 306 patients requiring randomization.

Interim analysis will be performed at hospital discharge of the first 102 and 204 randomized patients. If, at the time of these analyses, there is a trend toward better outcome in the control group (*p* < 0.1) the study will be terminated. The study will continue to randomize 306 patients if no adverse impact of the NAVA group is observed. The interim analysis will be performed at the coordinating center (Hospital Universitario Dr. Negrin, Las Palmas de Gran Canaria, Spain) by an independent Data and Safety Monitoring Board (DSMB). It is expected that DSMB meetings will be by conference call and DSMB discussions by email or conference call.

All adverse events reported will be sent to the DSMB as well as to the IRB for review (see Additional file 4). All serious events will be sent within 24 h after being received by the coordinating center. Nonserious events will be sent within 1 week of reception by the coordinating center. All unexpected, and related or possibly related, adverse events will be reported to the DSMB and the IRB. The DSMB will review the overall status of the study: number of patients enrolled overall and in each center, adherence to the protocol overall and by each center.

### Data analysis

Data will be collected in each participating ICU using a standardized form. Data will be transmitted to the coordinating center whenever a patient dies or is discharged from the hospital. Before exporting the data into a computerized data base at the coordinating center, a trained data collector from the coordinating center will check the completeness and the quality of information. Logical checks will be performed for missing data and to find inconsistencies, especially regarding clinical diagnosis, date, and ventilatory parameters. If necessary, the data collector will contact the investigator by phone to validate the data or reformat the data for entry into the database.

Descriptive statistics will be expressed as mean ± SD or median and interquartile range depending on the nature and distribution of the variables. Inferential statistics will use estimates of the mean of the differences and their 95 % confidence intervals (CI). Variables normally distributed will be compared with the Student’s *t* test. For variables without a normal distribution, the Mann-Whitney *U* rank test will be used for comparison. Categorical variables will be compared using Fisher’s exact test. The primary outcome variable (number of VFDs) will be assessed with the Student’s *t* test or the Mann-Whitney *U* rank test dependent on the distribution of the data. The relative risks and their 95 % CIs will be estimated. For all these comparisons, we will consider a difference to be statistically significant if *p* < 0.05.

## Discussion

Assisted MV is a highly complex process that requires an intimate interaction between the ventilator and the patient [25]. The complexity of the interaction between the ventilator and the patient with ARF is frequently underappreciated by the bedside clinician. The pattern of gas delivery by the ventilator and the patient’s own breathing pattern must match almost perfectly to avoid asynchrony between the patient and the ventilator. To date, we have no direct evidence that improving patient-ventilator synchrony improves patient outcome. However, there is growing evidence that asynchronies negatively correlate with clinical outcomes, including prolonged MV and ICU length of stay [1, 2] and lead to higher mortality [3].

NAVA is a newer mode for managing patients with ARF under MV. The insertion of the NAVA catheter is similar to the insertion of a nasogastric feeding tube and it is not associated with any additional risks. Patients with ARF who are able to breathe spontaneously while they are receiving MV are required to follow the lead of the ventilator and to adjust their respiratory center output to match the way the clinician sets the ventilator, otherwise, asynchrony will occur. Currently, it has become more accepted that the respiratory center of the patient in most circumstances is the most appropriate determinant of ventilatory pattern as a result of the increased recognition of the negative outcomes associated with patient-ventilator asynchrony [3]. In a recent study, Yonis et al. [26] reported reduced asynchrony during assisted ventilation with NAVA as compared to pressure support ventilation in 30 patients with respiratory failure who were randomly assigned to 23 h of pressure support ventilation and 23 h of NAVA. Also, in a previous study [27], we reported that NAVA, as compared to pressure support ventilation, resulted in improved synchrony, reduced ventilatory drive, increased breath-to-breath mechanical variability and improved patient comfort in 12 mechanically ventilated pediatric critically ill patients. In a series of 11 patients recovering from ARF, NAVA provided better patient-ventilator interaction [28]. In a recent study in 25 patients previously ventilated with controlled MV for at least 72 h who were randomized to be ventilated for 48 h with pressure support ventilation (*n* = 12) or NAVA (*n* = 13), NAVA improved diaphragmatic efficiency whereas pressure support ventilation did not [29].

Since NAVA does not control the patient’s ventilatory pattern, the patient is allowed to select whatever pattern the respiratory center considers appropriate. Neither pressure, flow, volume nor time is set by this novel ventilatory modality of assisted MV. All that is set during NAVA is the proportion of effort provided by the ventilator to supplement the patient’s own effort. In other words, NAVA follow the lead of the patient but does not force a ventilatory pattern on them [25]. If our hypothesis is correct, the introduction of NAVA into the routine care of adult patients with ARF will have a marked impact on the duration of MV, length of stay in critical care units, and health care costs. If our hypothesis is true, NAVA will become the preferred mode of assisted MV in patients spontaneously breathing with ARF.

### Trial status

The first patient was enrolled on 28 March 2014. The expected duration of the study is 50 months.

### Trial organization

The study principal investigators who contributed to the study design and approved the final protocol constitute the Steering Committee (Appendix 2). The Executive Committee comprises the main investigators of each participating center and is responsible for administrative, trial, and data management. The Data and Safety Monitoring Board is composed of three external, independent experts in critical care medicine, mechanical ventilation and ARDS and, with the general data provided by three internal members, it will recommend the continuation or discontinuation of the trial based on the data from the interim analysis. The Trial Management Team comprises a chief investigator, a project manager, a statistician, a clinical epidemiologist, and an investigator expert in clinical trials. The responsibilities of this team are:

1. Planning and conducting the study: designing the protocol, Case Report Forms, designing the investigator manual, and managing and controlling the data quality
2. Research center support: assisting the centers with the administrative submission, monitoring recruitment rates, providing sealed randomization envelopes, taking actions to increase patient enrollment, monitoring follow-up, auditing, and sending study materials to the research centers
3. Producing a monthly study newsletter (*Navanews*)
4. Programming a Research-in-Progress meeting at least once every year with the principal investigators from all sites
5. Statistical analysis and research reporting: interim and complete statistical analysis and helping to write the final manuscript

## Appendix 1

### NAVIATOR Network Investigators (in alphabetic order by hospitals)

Isabel Murcia (Hospital General de Albacete, Albacete, Spain); Javier Blanco, Luís Yuste, and María Carmen Espinosa (Hospital General de Ciudad Real, Ciudad Real, Spain); Carlos Ferrando, Marina Soro, Javier Belda, and José Ferreres (Hospital Clínico Universitario de Valencia, Valencia, Spain); Jesús Villar, Rosa Lidia Fernández, and Miguel Ángel García-Bello (Hospital Universitario Dr. Negrin, Las Palmas de Gran Canaria, Spain); César Pérez Calvo and Anxela Vidal (Hospital Universitario Fundación Jiménez Díaz, Madrid, Spain); Juan Alfonso Soler and Lucía Capilla (Hospital Universitario Morales Meseguer, Murcia, Spain); Raquel Montiel, Dácil Parrilla, Santiago Lubillo, and Lina Pérez-Méndez (Hospital Universitario NS de Candelaria, Santa Cruz de Tenerife, Spain); Francisco Alba and Ruth Corpas (Hospital General NS del Prado, Talavera de la Reina, Toledo, Spain); José Rubio (Hospital Puerta del Mar, Cádiz, Spain); David Pestaña, Nilda Martínez, Ángel Candela, and Pilar Cobeta (Hospital Universitario Ramón y Cajal, Madrid, Spain); Lorena Fernández, Jesús Blanco, César Aldecoa, and Jesús Rico (Hospital Universitario Río Hortega, Valladolid, Spain); Ana San Sebastián Hurtado and Marianela Hernández (Hospital Universitario de Txagorritxu, Vitoria, Spain); Domingo Martínez, Luis A. Conesa and Manuel Alfonso García (Hospital Universitario Virgen de Arrixaca, Murcia, Spain); José Manuel Añón, Elena González and Rosario Solano (Hospital Virgen de la Luz, Cuenca, Spain); María Mar Cruz-Acquaroni and María Ángela Magro-Martín (Hospital Virgen de la Salud, Toledo, Spain); Robert M Kacmarek (Massachusetts General Hospital, Boston, MA, USA).

## Appendix 2

Steering Committee: Jesús Villar, Javier Belda, Fernando Suárez-Sipmann, José Manuel Añón, Jesús Blanco, Lina Pérez-Méndez, and Robert M. Kacmarek.

Data and Safety Monitoring Board: Pedro de la Oliva, Paolo Pelosi, Lorenzo Berra (external members); Robert M Kacmarek, Rosa L Fernández, and Jesús Villar (internal members).

## Additional files

Additional file 1: Approval of the referral Ethics Committee and the Institutional Review Boards (IRB) of all participating hospitals. (PDF 4.14 mb)
Additional file 2: Informed consent form. (PDF 44 kb)
Additional file 3: SPIRIT checklist. (PDF 48 kb)
Additional file 4: Supplementary methods. (DOC 1486 kb)

## Abbreviations

AI: Asynchrony index
ARDS: Acute respiratory distress syndrome
ARF: Acute respiratory failure
COPD: Chronic obstructive pulmonary disease
DSMB: Data and Safety Monitoring Board
Edi: Electrical activity of the diaphragm
ICU: Intensive care unit
MDI: Metered-dose inhaler
MV: Mechanical ventilation
NAVA: Neurally Adjusted Ventilatory Assist
PaCO_2_: Partial pressure of carbon dioxide
PaO_2_/FiO_2_: Ratio between partial pressure of oxygen in arterial blood and fraction of inspired oxygen
PBW: Predicted body weight
PEEP: Positive end-expiratory pressure
PRVC: Pressure-regulated volume control
PS: Pressure support
SBT: Spontaneous breathing trial
SOFA: Sequential organ failure assessment
SpO_2_: Peripheral capillary oxygen saturation
VFD: Ventilator-free days
VS: Volume support
VT: Tidal volume
