# Supplementary material for: Neurally adjusted ventilatory assist in patients with acute respiratory failure: study protocol for a randomized controlled trial
Source: Trials. 2016 Oct 13;17:500. doi: 10.1186/s13063-016-1625-5 (PMC5064782; doi:10.1186/s13063-016-1625-5)
Supplement: Additional file 1: — Approval of the referral Ethics Committee and the Institutional Review Boards (IRB) of all participating hospitals. (PDF 4.14 mb) [file 13063_2016_1625_MOESM1_ESM.pdf]

GENERALITAT VALENCIANA
CONSELLERIA DE SANITAT

AGÈNCIA VALENCIANA DE SALUT

INFORME DEL COMITE ÉTICO DE INVESTIGACION CLINICA DEL HOSPITAL CLINIC UNIVERSITARI DE VALENCIA

D. Manuel Labiós Gómez, Secretario del Comité Ético de Investigación Clínica del Hospital Clínic Universitari de Valencia

CERTIFICA

Que en este Comité, en su reunión de ordinaria de fecha 29 de marzo de 2012, y según consta en el acta de la misma, se han analizado los aspectos éticos y científicos relacionados al proyecto de investigación que lleva por titulo:

Estudio comparativo, multicéntrico, aleatorizado, controlado, para evaluar la eficacia de la Asistencia Ventilatoria contralada neuralmente (NAVA) versus la Ventilación de Protección Pulmonar Convencional en pacientes con fallo respiratorio agudo

Que será llevado a cabo en el Servicio de Anestesiología y Reanimación y cuyo investigador principal es el Dr. Francisco Javier Belda Nacher, acordando que reúne las características adecuadas referentes a información a los pacientes y cumplimiento de los criterios éticos para la investigación médica y biomédica establecidos en la ***Declaración de Helsinki*** (Junio 1964, Helsinki, Finlandia) de la Asamblea Médica Mundial, y sus revisiones (Octubre 1975, Tokio, Japón), (Octubre 1983, Venecia, Italia), (Septiembre 1989, Hong Kong), (Octubre 1996, Somerset West, Sudáfrica), (Octubre 2000, Edimburgo) y (Octubre 2008 Seúl, Corea) en la ***Declaración Universal sobre el Genoma Humano y los Derechos del Hombre de la UNESCO*** y los acuerdos del ***Protocolo Adicional del Consejo de Europa para la protección de los Derechos del Hombre y de la dignidad del ser humano frente a la aplicaciones de la biología y de la medicina*** (París 12-1-1998, ratificado el 23-7-1999).

Lo que certifico a efectos oportunos.

Valencia, 29 de marzo de 2012.

Fdo. : Dr. D. Manuel Labiós Gómez
Secretario del Comité Ético de Investigación Clínica

GENERALITAT VALENCIANA
CONSELLERIA DE SANIDAD UNIVERSAL Y SALUD PÚBLICA

Hospital Clínic Universitari

DEPARTAMENT CLÍNIC MALVA-ROSA

Valencia, May 16th, 2016

To whom it may concern,

Dr. Antonio Peláez, as president of Valencia University Clinical Hospital Ethical Committee

CERTIFIES:

The project "A comparative, multicenter, randomized, controlled clinical trial of Neurally Ajusted Ventilatory Assist (NAVA) vs Conventional Lung Protective Ventilation in Patients with Acute Respiratory Failure", promoted by Dr. Jesús Villar from Hospital Dr. Negrin, was evaluated and approved in march 29th, 2012.

Dr. Antonio Peláez
President of Valencia University Clinical Hospital
Ethical Committee

482

ANEXO IV

INFORME DEL COMITÉ ÉTICO DE INVESTIGACIÓN CLÍNICA DE LA GERENCIA DE ATENCIÓN INTEGRADA DE ALBACETE Y SU ÁREA DE SALUD

D. Pedro Abizanda Soler, Presidente del Comité Ético de Investigación Clínica de la Gerencia de Atención Integrada de Albacete y su Área de Salud.

CERTIFICA

Que este Comité en su reunión de fecha 27 de abril de 2015, ha evaluado la propuesta del Promotor: **D. FRANCISCO JAVIER BLEDA NACHER**, para que se realice el Estudio con Productos Sanitarios, Código de Protocolo: **NAVA VS.**

TITULADO:

ESTUDIO COMPARATIVO, MULTICÉNTRICO, ALEATORIO, CONTROLADO PARA EVALUAR LA EFICACIA DE LA ASISTENCIA VENTILATORIA AJUSTADA NEURALMENTE (NAVA) FRENTE A LA VENTILACIÓN MECÁNICA PROTECTORA CONVENCIONAL EN EL MANEJO DE PACIENTES CON INSUFICIENCIA RESPIRATORIA AGUDA.

Y considera que:

Se cumplen los requisitos necesarios de idoneidad del protocolo en relación con los objetivos del estudio y están justificados los riesgos y molestias previsibles para el sujeto.

La capacidad de los investigadores y los medios disponibles son apropiados para llevar a cabo el estudio.

Son adecuados tanto el procedimiento para obtener el consentimiento informado como la compensación prevista para los sujetos por daños que pudieran derivarse de su participación en el estudio.

El alcance de las compensaciones económicas previstas no interfiere con el respeto a los postulados éticos.

Y que este Comité acepta que dicho estudio **Estudio con Productos Sanitarios** sea realizado en el Complejo Hospitalario Universitario de Albacete por la **Dra. Dª. Isabel Murcia Saez** como Investigadora Principal.

Que este Comité tanto en su composición como en los PNT´s cumple con las normas de BPC - CPMP/ ICH/ 135/ 95 del Real Decreto 223/2004 y que su composición actual es la siguiente:

Cod. 025861

[redacted]	[redacted]
**D. Pedro Abizanda Soler**	Presidente del CEIC. Licenciado en Medicina. Jefe de Sección de Geriatría.
**Dª. Mª Angeles Lloret Callejo**	Vicepresidenta del CEIC. Licenciada en Farmacia. Farmacéutica de Atención Primaria.
**Dª Carmen Díaz Delgado**	Secretaria del CEIC. Profesora Titular de Histología UCLM. Dra. Ciencias Médicas.
**D. Alberto Sánchez Romero**	Vocal del CEIC. Licenciado en Medicina. Especialista en Farmacología Clínica
**D. José Gerardo Espinosa Martínez**	Vocal del CEIC. Licenciado en Derecho. Ajeno a la profesión Sanitaria.
**Dª. Rosa Mª Blanco García**	Vocal del CEIC. Ajena a la profesión sanitaria
**D. Francisco Jesús Laserna Ibañez**	A.T.S del Servicio de Neurología del CHUA. Representante del Comité de Ética Asistencial.
**Dª. Eva García Martínez**	Vocal del CEIC. Licenciada en Farmacia Hospitalaria. CHUA
**D. Jesús López-Torres Hidalgo**	Vocal del CEIC. Coordinador de Formación e Investigación en la Gerencia de Atención Primaria.
**D. Javier Massó Orozco**	Vocal del CEIC. Licenciado en Medicina. Especialidad Medicina Familiar y Comunitaria.
**Dª Mª del Carmen Carrascosa Romero**	Vocal del CEIC. F.E.A de Pediatría. CHUA
**D. Manuel Gerónimo Pardo**	Vocal del CEIC. FEA Sección de Anestesiología y Farmacología Clínica
**Dª. Pilar Córcoles Jiménez**	Diplomada en Enfermería. Representante de la Comisión de Investigación en el C.E.I.C.
**Dª. Mª. Soledad Fernández de Córdoba**	Vocal del CEIC. F.E.A. Sección de Cirugía Pediátrica
**Dª Encarna Adrover Cebrián**	Vocal del CEIC. F.E.A. Oncología Médica CHUA
**D. Antonio Honguero Martínez**	Vocal del CEIC F.E.A. Cirugía Torácica. CHUA
**Dª Encarna Simarro Córdoba**	Vocal del CEIC Licenciada en Microbiología. CHUA
**Dª Syonghyun Nam Cha**	Vocal del CEIC. F.E.A. Anatomía Patológica. Representante del Biobanco.
**Dª María Pilar Marcos Rabal**	Vocal del CEIC Profesora Titular de Ciencias Morfológicas UCLM. Dra. Biología.
**D. Antonio Gutiérrez Díez**	Vocal del CEIC. FEA Sección de Cardiología
**Dª Karen Nieto Rodríguez**	Vocal del CEIC.FEA de Psiquiatría del CHUA
**D. Sergio Plata Paniagua. (oyente)**	Residente de Farmacia Hospitalaria

Lo que firmo en Albacete a 27 de abril de 2015.

Firmado:

D. PEDRO ABIZANDA SOLER

Cod. 025861

GERENCIA
DE ATENCIÓN INTEGRADA
DE ALBACETE
Hnos. Falcó, 37 - 02008 ALBACETE - Teléf.: 967 597 100

ANEXO V
CONFORMIDAD DE LA DIRECCION DEL CENTRO

D. IBRAHIM HERNANDEZ MILLAN, Director Gerente de la Gerencia de Atención Integrada de Albacete y vista la autorización del Comité Ético de Investigación Clínica,

CERTIFICA

Que conoce la propuesta formulada por el promotor D. Jesús Villar Hernández, para que se realice el Estudio de Investigación Clínica con Productos Sanitarios Código de protocolo "NAVA VS" " titulado: "Estudio comparativo, multicéntrico, aleatorio, controlado de Ventilación Asistida Ajustada Neuronalmente (NAVA) vs. Ventilación Mecánica Protectora Convencional en pacientes con insuficiencia respiratoria aguda por la Drª. D. Isabel Mª Murcia Sáez en calidad de Investigadora Principal.

Que está de acuerdo con el contrato firmado entre el Centro y el Promotor en el que se especifican todos los aspectos económicos de este Estudio de Investigación Clínica con Productos Sanitarios.

Que acepta la realización de dicho Estudio en este Centro.

Lo que firma en Albacete, a once de febrero de dos mil dieciséis.

Cod. 025861

Ciudad Real
Gerencia
Atención
Integrada

sescam
Servicio de Salud de Castilla-La Mancha

C/ Obispo Rafael Torija, s/n
Telf. 926 27 80 00 - Fax. 926 27 85 02
13005 CIUDAD REAL

INFORME DEL COMITÉ ETICO

El Comité Ético de Investigación Clínica del Hospital Universitario de General de Ciudad Real, en su reunión del día 24 de Febrero de 2015, Acta número 03/2015 con los asistentes:

Vicepresidente: Dr. José Luis del Burgo (Atención Primaria)
Secretaria: Dra. Esperanza Segura (Farmacología Clínica)
Vocales:
Dra. Montserrat Torromé (Farmacia Atención Primaria)
Dr. Alberto León Martín (Coordinador IDFyC)
Dr. José Luis Albasanz (Facultad Ciencias Químicas Ciudad Real)
Dª. Cinta Cumbreras Aguaded (Responsable Archivo y Documentación Clínica)
Dra. Otilia Santos (Farmacia Hospitalaria)
Dª. Mercedes Vidal (Lda. Farmacia Facultad de Enfermería)
D. José Medina (J. S. Gestión Económica)
D. Julián Rodríguez Almagro (DUE Servicio Urgencias)
Dra. Mª Isabel Porras Gallo (Facultad Medicina de Ciudad Real)

Acordó la:

Aprobación

Del Estudio: "**Estudio comparativo, multicéntrico, aleatorio, controlado para evaluar la eficacia de la asistencia ventilatoria ajustada neuralmente (NAVA) frente a la ventilación mecánica protectora convencional en el manejo de pacientes con Insuficiencia Respiratoria Aguda"**, con código de protocolo: NAVA VS, Promovido por, Grupo de Promoción Traslacional Dr. Jesús Villar, cuyo Investigador Principal es el Dr. Javier Blanco, el cual está previsto se realice en el Servicio de Medicina Intensiva del HGUCR.

Lo que firmo en Ciudad Real, a 25 de Febrero de 2015

HOSPITAL GENERAL CIUDAD REAL
COMITÉ ÉTICO DE INVESTIGACIÓN CLÍNICA

Fdo. Dra. Esperanza Segura Medina
Secretaria, C.E.I.C.

Castilla-La Mancha

Ciudad Real
Gerencia
Atención
Integrada

sescam
Servicio de Salud de Castilla-La Mancha

C/ Obispo Rafael Torija, s/n
Telf. 926 27 80 00 - Fax. 926 27 85 02
13005 CIUDAD REAL

INFORME DEL COMITÉ ÉTICO DE INVESTIGACIÓN CLÍNICA

Dª Esperanza Segura Molina, Secretaria del Comité Ético de Investigación Clínica del Hospital General Universitario de Ciudad Real,

CERTIFICA

Que este Comité ha evaluado la propuesta del Promotor Grupo de Promoción Traslacional Dr. Jesús Villar, para realizar la investigación del estudio titulado: **"Estudio comparativo, multicéntrico, aleatorio, controlado para evaluar la eficacia de la asistencia ventilatoria ajustada neuralmente (NAVA) frente a la ventilación mecánica protectora convencional en el manejo de pacientes con Insuficiencia Respiratoria Aguda"**, código de protocolo NAVA VS, en su reunión del día 24 de Febrero de 2015, Acta 03/2015 y en cuya discusión y aprobación final estuvieron presentes los siguientes miembros del C.E.I.C.:

Vicepresidente: Dr. José Luis del Burgo (Atención Primaria)
Secretaria: Dra. Esperanza Segura (Farmacología Clínica)
Vocales:
Dra. Montserrat Torromé (Farmacia Atención Primaria)
Dr. Alberto León Martín (Coordinador IDFyC)
Dr. José Luis Albasanz (Facultad Ciencias Químicas Ciudad Real)
Dª. Cinta Cumbreras Aguaded (Responsable Archivo y Documentación Clínica)
Dra. Otilia Santos (Farmacia Hospitalaria)
Dª. Mercedes Vidal (Lda. Farmacia Facultad de Enfermería)
D. José Medina (J. S. Gestión Económica)
D. Julián Rodríguez Almagro (DUE Servicio Urgencias)
Dra. Mª Isabel Porras Gallo (Facultad Medicina de Ciudad Real)

y que considera que:

Se cumplen los requisitos necesarios de idoneidad del protocolo en relación con los objetivos de estudios y están justificados los riesgos y molestias previsibles para el sujeto.

La capacidad del investigador y los medios disponibles con apropiados para llevar a cabo el estudio.

Son adecuados tanto el procedimiento para obtener el consentimiento informado, como la compensación prevista para los sujetos, por los daños que pudieran derivarse de su participación en el ensayo.

Ciudad Real
Gerencia
Atención
Integrada

sescam
Servicio de Salud de Castilla-La Mancha

C/ Obispo Rafael Torija, s/n
Telf. 926 27 80 00 - Fax. 926 27 85 02
13005 CIUDAD REAL

El alcance de las compensaciones económicas previstas no interfiere con respecto a los postulados éticos.

Y que el Comité acepta que dicha investigación clínica sea realizada en el Hospital General Universitario de Ciudad Real por el Dr. Javier Blanco del Servicio de Medicina Intensiva como investigador principal.

Lo que firmo en Ciudad Real, a 25 de Febrero de 2015

Hospital ... CIUDAD REAL
COMITÉ ÉTICO DE INVESTIGACIÓN CLÍ...

Fdo: Dra. Esperanza Segura Molina
Secretaria C.E.I.C.

Dr. D. Javier Bécares Martínez, Secretario del COMITE ÉTICO DE INVESTIGACION CLINICA DE LA FUNDACION JIMENEZ DIAZ.

CERTIFICA:

Que este Comité ha evaluado la propuesta del promotor para que se realice el ensayo clínico:

Promotor: Dr. Jesús Villar Hernández
Código de protocolo: NA
Nº Eudract: NA
Título: "Estudio comparativo, multicéntrico, aleatorio, controlado para evaluar la eficacia de la asistencia ventilatoria ajustada neuralmente (NAVA) frente a la ventilación mecánica protectora convencional en el manejo de pacientes con Insuficiencia Respiratoria Aguda"
Versión del protocolo: 01.0 de 10 de enero de 2012
Versión de la Hoja de Información al Paciente: 2 de abril de 2013

Con el producto sanitario NAVA y considera que,

- Se cumplen los requisitos necesarios de idoneidad del protocolo en relación con los objetivos del estudio y están justificados los riesgos y molestias previsibles para el sujeto.
- La capacidad del investigador y los medios disponibles son apropiados para llevar a cabo el estudio.
- Son adecuados tanto el procedimiento para obtener el consentimiento informado, como la compensación prevista para los sujetos por daños que pudieran derivarse de su participación en el ensayo.
- El alcance de las compensaciones económicas previstas no interfiere con el respeto a los postulados éticos.

Por tanto, este CEIC como Comité de referencia, habiendo tenido en cuenta los dictámenes de los CEICs implicados, acepta que dicho ensayo clínico sea realizado por los siguientes investigadores en los respectivos centros:

Dr. César Pérez Calvo / Fundación Jiménez Díaz / Madrid

Lo que firmo en Madrid a 13 de mayo de 2013

FUNDACION JIMENEZ DIAZ COMITE ETICO DE INVESTIGACION CLINICA CEIC - FJD MADRID

Dr. Javier Bécares Martínez
Secretario CEIC-FJD

Avda. de los Reyes Católicos, 2 - 28040-Madrid
Teléfono: 91 5443720 – FAX: 915498075 – e-mail: ceic@fjd.es

ENSAYO CLINICO

Promotor: Dr. Jesús Villar Hernández
Código de protocolo: NA
Nº Eudract: NA
Título: "Estudio comparativo, multicéntrico, aleatorio, controlado para evaluar la eficacia de la asistencia ventilatoria ajustada neuralmente (NAVA) frente a la ventilación mecánica protectora convencional en el manejo de pacientes con Insuficiencia Respiratoria Aguda"
Versión del protocolo: 01.0 de 10 de enero de 2012
Versión de la Hoja de Información al Paciente: 2 de abril de 2013

Dr. D. Javier Bécares Martínez, Secretario del COMITE ÉTICO DE INVESTIGACION CLINICA DE LA FUNDACION JIMENEZ DIAZ.

HACE CONSTAR QUE:

1º En la reunión celebrada el día 26 de febrero de 2013 se evaluó el ensayo clínico de referencia -acta nº 02/13- y, una vez aceptadas las aclaraciones solicitadas, se decidió emitir el informe favorable. .
2º En dichas reuniones se cumplieron los requisitos establecidos en la legislación vigente –Real decreto 223/2004 y Decreto 39/94 de la CAM- para que la decisión del citado CEIC sea válida.
3º El CEIC-FJD, tanto en su composición, como en los PNT cumple con las normas BPC
4º La composición actual del CEIC-FJD es la siguiente:

Dra. Belen Acevedo Marín. *Médico Asistencial*
Dra. Mª José Almodóvar Carretón. Farmacéutica de Atención Primaria
Dra. Carmen Ayuso García. *Médico Asistencial. Miembro Comité de Investigación.*
Dr. Javier Bécares Martínez. ***(Secretario)****. Farmacéutico de la FJD*
Dra. Miriam Blanco Rodríguez. *Médico Asistencial*
Dra. Macarena Bonilla Porras. ***(Vicepresidenta).*** *Farmacéutico de la FJD*
Dr. Emilio Calvo Crespo. **(Presidente)** *Médico Asistencial.*
Dña. Teresa Castillo Sánchez. DUE.
Dra. Isabel Egocheaga Cabello. *Médico de Atención Primaria*
Dr. Ricardo Fernández Roblas. *Médico Asistencial*
Dr. Yann Izarzugaza Peron. *Médico Asistencial*
Dr. Alberto Lendinez Fornis. *Médico Asistencial*
D. Jose Angel Martínez Peláez. *Lego no vinculado a la Institución*
Dra. Dolores Martínez Pérez. *Médico Asistencial*
D. Manuel Matamoros Fernández. *Lego no vinculado a la Institución*
Dra. Maria Rosario Noguero Meseguer. *Médico Asistencial*
Dr. Germán Peces Barba Romero. *Médico Asistencial*, *Miembro Comité de Investigación*
D. Luis Ortega Alba. *Abogado*
Dr. Mauro Javier Oruezabal Moreno. *Médico Asistencial*
Dr. Francisco Javier Ruiz Hornillos. *Médico Asistencial y Miembro Comité Ética Asistencial*.
Dra. Olga Sánchez Pernaute. Médico Asistencial
Dra. Aranzazu Sancho López. *Farmacóloga Clínico*

Para que conste donde proceda, y a petición del promotor,

Lo que firmo en Madrid a 13 de mayo de 2013

FUNDACION JIMENEZ DIAZ
COMITE ETICO DE INVESTIGACION CLINICA
CEIC - FJD
MADRID

Dr. Javier Bécares Martínez
Secretario CEIC-FJD

Avda. de los Reyes Católicos, 2 - 28040-Madrid
Teléfono: 91 5443720 – e-mail: ceic@fjd.es

Area Vega Media del Segura

Servicio Murciano de Salud

Informe Dictamen Protocolo Favorable
EECC con Medicamento – Unicéntrico/Local

C.P. NAVA VS - N.E. - - - C.I. EN-12/12

31 de Mayo de 2012

Dra. María Dolores Nájera Pérez
Presidenta del CEIC Hospital General Universitario José María Morales Meseguer

CERTIFICA

Que el CEIC Hospital General Universitario José María Morales Meseguer en su reunión del día **31/05/2012**, acta **EXTRAORDINARIA,** ha evaluado la propuesta del promotor referida al estudio:

Título: Estudio comparativo, multicéntrico, randomizado de la Ventilación Asistida Ajustada Neuronalmente (NAVA) vs. Ventilación Convencional Protectora del Pulmón en Pacientes con Fallo Respiratorio Agudo.

Código Promotor: NAVA VS **Nº EUDRACT:** - - **Código Interno:** EN-12/12
Promotor: Unidad Investigación, Hospital Universitario Dr. Negrín
Monitor/CRO: Grupo de Investigación Traslacional Dr. J. Villar
Versión Protocolo Evaluada: 04-05-2012
Versión Hoja Información al Paciente Evaluada: GENERAL / 04-05-2012
Fecha Entrada: 04/05/2012

1º. Considera que:
- El ensayo se plantea siguiendo los requisitos del Real Decreto 223/2004, de 6 de febrero y las normas que lo desarrollan, y su realización es pertinente.
- Se cumplen los requisitos necesarios de idoneidad del protocolo en relación con los objetivos del estudio y están justificados los riesgos y molestias previsibles para el sujeto.
- Son adecuados tanto el procedimiento para obtener el consentimiento informado como la compensación prevista para los sujetos por daños que pudieran derivarse de su participación en el ensayo.
- El alcance de las compensaciones económicas previstas no interfiere con el respeto a los postulados éticos.
- La capacidad del investigador y sus colaboradores, y las instalaciones y medios disponibles, tal y como ha sido informado, son apropiados para llevar a cabo el estudio.

2º. Por lo que este CEIC emite un **DICTAMEN FAVORABLE**.

3º. Este CEIC acepta que dicho ensayo sea realizado en los siguientes CEIC/Centros por los Investigadores:

CEIC Hospital General Universitario José María Morales Meseguer	(C) Dr. Juan Alfonso Soler Barnes (Medicina Intensiva) Hospital General Universitario J.M. Morales Meseguer

Lo que firmo en Murcia, a31 de Mayo de 2012

Dra. María Dolores Nájera Pérez
Presidenta del CEIC Hospital General Universitario José María Morales Meseguer

Hospital General Universitario J.M. Morales Meseguer
Marqués de los Vélez s/n Murcia 30008 Murcia España
Tel. 968 36 52 02 Fax. 968 36 09 49 Correo electrónico mariad.najera@carm.es

M.M.12

"Hospital Morales Meseguer" – C/. Marqués de los Vélez, s/n • Teléf. 968 36 09 00 • Fax. 968 23 24 84 • 30008 MURCIA

Servicio Canario de la Salud
HOSPITAL UNIVERSITARIO
NTRA. SRA. DE CANDELARIA

COMITÉ ÉTICO DE INVESTIGACIÓN CLÍNICA
ENTRADA / SALIDA
Nº 38 FECHA: 4/02/13

Gobierno de Canarias

INMACULADA PLASENCIA GARCIA, Secretario/a del **COMITÉ ÉTICO DE INVESTIGACIÓN CLÍNICA**

CERTIFICA

Que este Comité ha evaluado la propuesta del Investigador Principal del Proyecto de Investigación Biomédica:

CÓDIGO: PI-02/13
INVESTIGADOR: SANTIAGO LUBILLO MONTENEGRO

Proyecto de Investigación Biomédica: Estudio comparativo, multicéntrico, aleatorizado, controlado para evaluar la eficacia de la asistencia ventilatoria ajustada neuralmenta (NAVA) frente a la ventilación mecánica protectora convencional en el manejo de pacientes con insuficiencia respiratoria aguda (JESÚS VILLAR HERNÁNDEZ)

y considera que:

- Se cumplen los requisitos necesarios de idoneidad del protocolo en relación con los objetivos del estudio y están justificados los riesgos y molestias previsibles para el sujeto, teniendo en cuenta los beneficios esperados.

- Son adecuados tanto el procedimiento para obtener el consentimiento informado

- La capacidad del investigador y sus colaboradores, y las instalaciones y medios disponibles, tal y como ha sido informado, son apropiados para llevar a cabo el estudio.

Este CEIC, resuelve **Aprobado** que dicho estudio sea realizado en el Hospital Universitario Nuestra Señora de Candelaria, actuando como investigador principal el Dr./Dra. SANTIAGO LUBILLO MONTENEGRO.

Lo que firmo en Santa Cruz de Tenerife, a 04 de febrero de 2013

Secretario/a del CEIC

INMACULADA PLASENCIA GARCIA

Virginia Arroyo Pineda, Secretaria del Comité Ético de Investigación Clínica del Área Integrada de Salud de Talavera de la Reina,

CERTIFICA

Que este Comité en las reuniones celebradas entre los días 19 de febrero y 16 de abril de 2.013 ha evaluado la propuesta referida al estudio:

- **Título:** Estudio NAVA. Comparativo, multicéntrico, aleatorio de Ventilación Asistida Ajustada Neuronalmente *vs* ventilación protectora convencional en pacientes con insuficiencia respiratoria aguda.
- **Protocolo nº:** NAVA.
- **Promotor:** Jesús Villar Hernández.
- **Tipo de estudio:** Ensayo clínico para evaluar la eficacia de una intervención sanitaria.
- **Investigador en el área**: Dr. Francisco Alba (Medicina Intensiva de la UCI).

Considera que:

- Se cumplen los requisitos necesarios de idoneidad del proyecto en relación con los objetivos del mismo.
- Que la capacidad del investigador y los medios disponibles son apropiados para llevar acabo el estudio.
- Que no interfiere los postulados éticos y respeta los derechos fundamentales de la persona.

Por lo que emite un **DICTAMEN FAVORABLE**.

Lo que firmo en Talavera de la Reina, a 16 de abril de 2.013

-Servicio de Salud de Castilla la Mancha- sescam CEIC Área Sanitaria de Talavera de la Reina

Fdo: Virginia Arroyo Pineda,
Secretaria del CEIC

Área de Gestión Integrada de Salud de Talavera de la Reina
INFORME DEL COMITÉ ÉTICO DE INVESTIGACIÓN CLÍNICA

Virginia Arroyo Pineda, Secretaria del Comité Ético de Investigación Clínica del Área Integrada de Salud de Talavera de la Reina, hace constar que:

- En la reunión ordinaria celebrada el día 16 de abril de 2013, se decidió emitir el informe correspondiente al estudio indicado en la página anterior.
- En dicha reunión se cumplieron los requisitos establecidos en la legislación vigente para que la decisión del citado CEIC sea válida.
- La composición del CEIC que evaluó el ensayo fue la siguiente:

Presidenta	**Julia Díez Izquierdo**<br>Esp. Cirugía General y del Aparato Digestivo<br>Hospital Ntra. Sra. del Prado
Vicepresidente	**Antonio Villar Ramos**<br>Ldo. Derecho<br>Hospital Ntra Sra del Prado
Secretaria	**Virginia Arroyo Pineda**<br>Farmacéutica de Atención Primaria<br>Hospital Ntra. Sra. del Prado
Vocales	**Mª Antonia Berrocal Javato**<br>Esp. Farmacia Hospitalaria<br>Hospital Ntra. Sra. del Prado<br>**Mª Antonia Crespo Panadero**<br>Enfermera<br>Hospital Ntra. Sra. del Prado<br>**Carlos Fernández Hernández**<br>Apoyo administrativo<br>Hospital Ntra Sra del Prado<br>**Mª Rocío Gil Ruiz**<br>Esp. Oftalmología<br>Hospital Ntra. Sra. del Prado<br>**Maria López Gómez**<br>Esp. Anestesiología y Reanimación<br>Hospital Nuestra Señora del Prado<br>**Rosa Esteban García**<br>Enfermera<br>Hospital Ntra. Sra. del Prado<br>**Juan Ángel Pérez Andújar**<br>Esp. Cirugía General y del Ap. Digestivo<br>Hospital Ntra. Sra. del Prado<br>**Begoña Polonio López**<br>Psicóloga<br>Decana de la Univ. de Castilla-La Mancha<br>**Esperanza Segura Molina**<br>Farmacóloga<br>Hospital General de Ciudad Real

Lo que firmo en Talavera de la Reina, a 16 de abril de 2013.

Fdo. Virginia Arroyo Secretaria del CEIC

Hospital Nuestra Señora del Prado - Ctra. Nacional V, km. 114
Talavera de la Reina 45600 Toledo España
Tel. (925) 80 36 00 Ext. 86.316; varroyo@sescam.org

D. ª MONICA SALDAÑA VALDERAS COMO SECRETARIA DEL COMITÉ DE ETICA DE LA INVESTIGACION DEL HOSPITAL UNIVERSITARIO PUERTA DEL MAR Y DISTRITO BAHIA DE CADIZ LA JANDA.

HACE CONSTAR:

Que en su reunión de fecha 26 de marzo de 2015 se ha revisado el estudio de investigación **promovido** por el **Dr. Jesús Vilar Hernández** del cual es **Investigador Principal** en nuestro centro el **Dr. José Rubio Quiñones y** titulado:

Estudio comparativo, multicéntrico, randomizado de la ventilación asistida ajustada neuronalmente (NAVA) vs. Ventilación convencional protectora del pulmón en pacientes con fallo respiratorio agudo.

Y hace constar que el citado proyecto es viable,

Que presenta suficiente rigor metodológico,

Que la evaluación de costes económicos es correcta,

Que con respecto a su vertiente ética el proyecto cumple los requisitos necesarios de idoneidad del protocolo en relación con los objetivos del estudio.

Y para que así conste, firmo la presente en Cádiz a 30 de marzo de 2015

CONSEJERÍA DE SALUD
Servicio Andaluz de Salud
JUNTA DE ANDALUCIA
Q-9150013-B

C.S.7

CONFORMIDAD DE LA
DIRECCIÓN DEL CENTRO

D. Rafael Pereiro Hernández, Director Gerente del Hospital Universitario "Puerta del Mar" de Cádiz y vista la aprobación del Comité de Ética de la Investigación

C E R T I F I C A

Que conoce la propuesta realizada por el **Promotor** el **Dr. Jesús Vilar Hernández** para que sea realizado en este Centro el estudio de investigación del cual es **Investigador Principal** el **Dr. José Rubio Quiñones y** titulado:

Estudio comparativo, multicéntrico, randomizado de la ventilación asistida ajustada neuronalmente (NAVA) vs. Ventilación convencional protectora del pulmón en pacientes con fallo respiratorio agudo.

Que acepta la realización de dicho estudio en este Centro.

Lo que firma en Cádiz, a 30 de marzo de 2015

CONSEJERÍA DE SALUD
Servicio Andaluz de Salud
JUNTA DE ANDALUCIA
Dirección Gerencia
Hospital Univ. Puerta del Mar
Q- 9150013-B

C.S.7

INFORME DEL COMITÉ ETICO DE INVESTIGACION CLINICA

Dra. ITZIAR DE PABLO LOPEZ DE ABECHUCO. Secretaria del Comité Ético de Investigación Clínica del Hospital Universitario Ramón y Cajal.

CERTIFICA

Que el Comité Ético de Investigación Clínica ha evaluado la propuesta del Promotor: **Dr. Jesús Villar Hernández del Hospital Universitario Dr. Negrín del Servicio Canario de Salud** para que sea realizado en este Hospital el Ensayo Clínico no ajustado a R.D. 223/04 con código de Protocolo: **NAVA** titulado:

Estudio comparativo, multicéntrico, aleatorio, controlado para evaluar la eficacia de la asistencia ventilatoria ajustada neuralmente (NAVA) frente a la ventilación mecánica protectora convencional en el manejo de pacientes con Insuficiencia Respiratoria Aguda

Protocolo versión 01.0 de fecha 10/enero/2012
Hoja de información para el familiar/representante legal versión 01.0 de fecha 10/enero/2012

Se cumplen los requisitos necesarios de idoneidad del Protocolo en relación con los objetivos del estudio y están justificados los riesgos y molestias previsibles para el sujeto.

La capacidad del Investigador y los medios disponibles son apropiados para llevar a cabo el estudio.

Son adecuados el procedimiento previsto para obtener el consentimiento informado y la compensación prevista para los sujetos por daños que pudieran derivarse de su participación en el ensayo.

El alcance de las compensaciones económicas previstas no interfieren con el respeto a los postulados éticos.

Y que este Comité acepta que dicho Ensayo Clínico sea realizado en este Hospital por el **Dr. David Pestañas Lagunas** como investigador Principal.

Lo que firmo en Madrid a 18 de octubre de 2012

Hospital Universitario Ramón y Cajal
SaludMadrid
COMITÉ ÉTICO DE INVESTIGACIÓN CLÍNICA

Fdo.: Itziar de Pablo López de Abechuco
Secretaria CEIC

Dra. Itziar de Pablo López de Abechuco, Secretaria del Comité Ético de Investigación Clínica (CEIC)

del HOSPITAL UNIVERSITARIO RAMÓN Y CAJAL

HACE CONSTAR QUE:

1º En la reunión del 28 de mayo de 2012 se evaluó el Ensayo Clínico no ajustado a R.D. 223/04, con código de protocolo: **NAVA**

2º En dicha reunión se cumplieron los requisitos establecidos en la legislación vigente.

3º El CEIC, tanto en su composición, como en los PNT se ajusta a las normas de BPC.

4º La composición del CEIC del Hospital Ramón y Cajal en la citada fecha, era la siguiente:

- Dra. Mª Jesús Blanchard Rodríguez (Hematología).
- Dra. Itziar de Pablo López de Abechuco. (Farmacóloga Clínica).
- Dr. Alejandro del Río Busto (Cardiología).
- Dra. María de los Ángeles Gálvez Múgica. (Farmacóloga Clínica).
- Dr José Manuel Garrido Jiménez (Cirugía Cardiovascular).
- Dra. Carmen Guillén Ponce (Oncología Médica).
- Dra Elena Molina Martín (Servicio de Farmacia Área 4 A. Primaria).
- D. Alfonso Muriel García (Bioestadística Clínica).
- Dr José Antonio Pérez Molina (Enfermedades Infecciosas)
- Dra. Cristina Pueyo López. (Licenciada en Farmacia y Master de Bioética).
- D Cristóbal Francisco Rodríguez Martín. (DUE Psiquiatría).
- Dña. Carmen Ruiz Ballesteros. (Licenciada en Derecho. No vinculada laboralmente al Hospital).
- D. Javier Sánchez Casajús (Licenciado en Económicas. No vinculado laboralmente al Hospital)
- Dr José Miguel Sánchez Torres (Oncología MD Anderson).
- Dra Sonsoles Sancho García. (Oncología Radioterápica).
- Dr José Luis San Millán López (Genética Molecular).
- Dra. Sonia Soto Díaz (Médico de Familia, Atención Primaria).

Según nuestros PNTs, en las evaluaciones de los protocolos en los que algún miembro del CEIC actúa como investigador/colaborador, éste se ausenta de la reunión durante la discusión del mismo.

Para que conste donde proceda, y a petición del promotor,

Madrid 18 de octubre de 2012.

Fdo. Dra. Itziar de Pablo López de Abechuco
Secretaria del CEIC

HOSPITAL UNIVERSITARIO
RÍO HORTEGA

Sacyl
SANIDAD DE CASTILLA Y LEÓN

INFORME DEL COMITÉ ÉTICO DE INVESTIGACIÓN CLÍNICA

D. FLORENTINO PINACHO PELAEZ, Secretario del Comité Ético de Investigación Clínica del Hospital Universitario Río Hortega de Valladolid Oeste.

CERTIFICA:

Que este Comité ha evaluado, en su reunión del día 30 de Octubre de 2013, la Enmienda Relevante del Estudio titulado: **"Estudio comparativo, multicéntrico, aleatorio, controlado para evaluar la eficacia de la asistencia ventilatoria ajustada neuralmente (NAVA) frente a la ventilación mecánica protectora convencional en el manejo de pacientes con Insuficiencia Respiratoria Aguda"** y considera que:

Se cumplen los requisitos necesarios de idoneidad del protocolo en relación con los objetivos del estudio y están justificados los riesgos y molestias previsibles para el sujeto.

La capacidad del investigador y los medios disponibles son apropiados para llevar a cabo el Estudio.

Y que este Comité acepta que dicho Estudio sea realizado en el Hospital Universitario del Río-Hortega por el **Dr. Jesús Blanco Varela y Dr. Cesar Aldecoa** como Investigadores Principales

Lo que firmo en Valladolid, a 06 de Noviembre de 2013

Sacyl
HOSPITAL UNIVERSITARIO
"RIO HORTEGA"
COMITÉ ÉTICO DE
INVESTIGACIÓN CLÍNICA

Fdo. D Florentino Pinacho Peláez
Secretario CEIC

Junta de
Castilla y León

CEIC E

EUSKO JAURLARITZA
GOBIERNO VASCO

OSASUN SAILA
DEPARTAMENTO DE SALUD

DICTAMEN DEL COMITE ETICO DE INVESTIGACION CLINICA DE EUSKADI (CEIC-E)

Dra. Iciar Alfonso Farnós como Secretaria del CEIC Comunidad Autónoma de País Vasco (CEIC-E)

CERTIFICA

Que este Comité, que reúne los requisitos establecidos en el R.D 223/2004, de 6 de Febrero por el que se regulan los ensayos clínicos con medicamentos, ha evaluado la propuesta del promotor: Dª Marianela Hernández López (Cuidados Intensivos) Hospital Universitario de Araba (sede Txagorritxu) , para que se realice el ensayo clínico Código Promotor: NAVA, de Título: **Estudio comparativo, multicéntrico, aleatorio de Ventilación Asistida Ajustada Neuronalmente (NAVA) vs ventilación protectora convencional en pacientes con insuficiencia respiratoria aguda**

Versión del Protocolo: 01.1; December, 18, 2012
Versión Hoja Información al Paciente y Consentimiento Informado: ENERO 2013

Considera que,

Se cumplen los requisitos necesarios de idoneidad del protocolo en relación con los objetivos del estudio y están justificados los riesgos y molestias previsibles para el sujeto.

La capacidad de los Investigadores y los medios disponibles son apropiados para llevar a cabo el estudio.

Son adecuados tanto el procedimiento para obtener el consentimiento informado como la compensación prevista para los sujetos por daños que pudieran derivarse de su participación en el ensayo.

El alcance de las compensaciones económicas previstas no interfiere con el respeto a los postulados éticos.

Este Comité Ético de Investigación Clínica, tanto en su composición como en los Procedimientos normalizados de Trabajo, cumple con las normas de buena práctica clínica (CPMP/ ICH/135/95) conforme a lo establecido en el capítulo VIII del Real Decreto 223/2004, de 6 de Febrero.

Y que este Comité reunido el día 30 de enero de 2013 (recogido en acta 01/2013) ha decidido emitir dictamen favorable a que dicho ensayo sea realizado en:

- Dª Marianela Hernández López (Cuidados Intensivos) Hospital Universitario de Araba (sede Txagorritxu)

Lo que firmo en Vitoria-Gasteiz, a 14 de febrero de 2013

Fdo:

P. A.

Dra. Iciar Alfonso Farnós
Secretaria del CEIC Comunidad Autónoma de País Vasco (CEIC-E)

GOBIERNO VASCO
OSASUN ETA KONTSUMO SAILA
[illegible] SANIDAD Y CONSUMO
14 FEB 2013
[illegible] Batzorde Etikoa
[illegible] de Investigación Clínica de Euskadi (CEIC-E)

Servicio Murciano de Salud

Arrixaca
Hospital Universitario "Virgen de la Arrixaca"
Ctra. Madrid - Cartagena • Telf. 968 36 95 00
30120 El Palmar (Murcia)

Dr. D. Antonio Piñero Madrona
Presidente del CEIC Hospital Virgen de la Arrixaca

CERTIFICA

Que el CEIC Hospital Virgen de la Arrixaca en su reunión del día 25/02/2013, acta 02/13 ha evaluado la propuesta del promotor referida al estudio:

Título: Estudio comparativo, multicéntrico, aleatorio de Ventilación Asistida Ajustada Neuronalmente (NAVA) vs ventilación protectora convencional en pacientes con insuficiencia respiratoria aguda

Código Promotor: NAVA

1º. Considera que

- El ensayo se plantea siguiendo los requisitos de la Ley 14/2007, de 3 de julio, de Investigación Biomédica y su realización es pertinente.
- Se cumplen los requisitos necesarios de idoneidad del protocolo en relación con los objetivos del estudio y están justificados los riesgos y molestias previsibles para el sujeto.
- Son adecuados tanto el procedimiento para obtener el consentimiento informado como la compensación prevista para los sujetos por daños que pudieran derivarse de su participación en el estudio.
- El alcance de las compensaciones económicas previstas no interfiere con el respeto a los postulados éticos.
- La capacidad de los Investigadores y los medios disponibles son apropiados para llevar a cabo el estudio.

2º. Por lo que este CEIC emite un **DICTAMEN FAVORABLE** y acepta que sea realizado por el Dr**. Domingo Martínez Baño** en el Hospital Clínico Universitario Virgen de la Arrixaca.

Lo que firmo en Murcia, a 25 de febrero de 2013

Fdo:

Servicio Murciano de Salud
Hospital "Virgen de la Arrixaca"
Comite Etico de Investigación Clínica
MURCIA

Dr. D. Antonio Piñero Madrona
Presidente del CEIC Hospital Virgen de la Arrixaca

Mod. 141

HOSPITAL VIRGEN DE LA LUZ CUENCA
Hermandad Donantes de Sangre, 1
Teléf. 969 179 900 / Fax 969 230 407
16002 CUENCA

sescam
Servicio de Salud de Castilla-La Mancha

Informe del Comité Ético de Investigación Clínica

Presentado a este Comité, el proyecto de investigación denominado **"Estudio comparativo, multicéntrico, aleatorio, controlado para evaluar la eficacia de la asistencia ventilatoria ajustada neuralmente (NAVA) frente a la ventilación mecánica protectora convencional en el manejo de pacientes con Insuficiencia Respiratoria Aguda"** Cuyo investigador Principal: Dr. José Manuel Añón Elizalde, para su evaluación desde su punto de vista ético, una vez estudiados y valorados el objeto y procedimiento seguido, lo consideramos **Conforme**, al observar el cumplimiento de los principios básicos que han de regir cualquier investigación en seres humanos,

Lo que firmo a Cuenca, 20 de junio de 2012

LA PRESIDENTE DEL COMITÉ ÉTICO DE INVESTIGACIÓN CLÍNICA

P.O. Secretaria CEIC.

Fdo.: Dra. Mª José Peirón Puyal.

Comité Etico
de Investigación Clínica
AREA DE SALUD DE CUENCA

COMPLEJO HOSPITALARIO DE TOLEDO
HOSPITAL VIRGEN DE LA SALUD
Avda. Barber, 30. 45004. Toledo. Teléfono 925 269200

sescam
Servicio de Salud de Castilla-La Mancha

C.E.I.C. SALIDA
FECHA: 13/12/2012
N.º 106

DICTAMEN DEL COMITÉ ETICO DE INVESTIGACIÓN CLÍNICA

D. Fernando Jiménez Torres, Secretario del Comité Ético de Investigación clínica del "Complejo Hospitalario de Toledo".

CERTIFICA:

Que este Comité, en su reunión de fecha 28 de noviembre de 2012, ha evaluado la propuesta del promotor: D. Jesus Villar - U. Investigacion - H. U. Dr. Negrin - Las Palmas de Gran Canaria, en relación a la evaluación del Estudio.

Título: “ESTUDIO COMPARATIVO, MULTICENTRICO, RANDOMIZADO DE LA VENTILACION ASISTIDA AJUSTADA NEURONALMENTE (NAVA)vs. VENTILACION CONVENCION PROTECTORA DEL PULMON EN PACIENTES CON FALLO RESPIRATORIO AGUDO”.

Este Comité emite **DICTAMEN FAVORABLE** para que se lleve a cabo el **Estudio** en el Hospital Virgen de la Salud de Toledo, siendo la investigadora principal la **Dra. Maria del Mar Cruz Acquaroni. S°. U.C.I** del Complejo Hospitalario de Toledo.

Que el Comité tanto en su composición como en los PNT cumple con las normas de BPC (CPMP/ICH/135/95) y con el Real Decreto 223/2004, y su composición actual es la siguiente:

Presidente:	Dr. Antonio Gómez Rodriguez
Vicepresidente:	D. David García Marco
Secretario:	Dr. Fernando Jiménez Torres
Vocales:	Dra. Enriqueta Muñoz Platón
	Dña. Manuela Martínez Camacho
	Dña. María Antonia Mareque Ortega
	Dr. Juan Carvajal Alonso
	Dr. Rafael Cuena Boy
	Dña. Mª Ángeles Jiménez Navarro
	Dr. Luis Fernando Viejo LLorente
	Dña. Macarena González Villamor
	Dña. Elena Carrascoso Sánchez
	Dr. Jose Gil Sales
	Dña. Mª José Espinosa de los Monteros Garde
	Dña. Inés Martínez Galán
	Dr. Jesús Santos Del Cerro
	Dña. Alica Hanzeliková Pogrányivá

Que en dicha reunión del Comité Ético de Investigación Clínica se cumplió el quórum perceptivo legalmente.

Lo que firmo en Toledo, 28 de noviembre de 2012.

COMPLEJO HOSPITALARIO DE TOLEDO
COMITE ETICO DE INVESTIGACION CLINICA

Fdo.: Fernando Jiménez Torres
