# Supplementary material for: Neurally adjusted ventilatory assist in patients with acute respiratory failure: study protocol for a randomized controlled trial
Source: Trials. 2016 Oct 13;17:500. doi: 10.1186/s13063-016-1625-5 (PMC5064782; doi:10.1186/s13063-016-1625-5)
Supplement: Additional file 2: — Informed consent form. (PDF 44 kb) [file 13063_2016_1625_MOESM2_ESM.pdf]

HOJA DE INFORMACIÓN PARA EL FAMILIAR / REPRESENTANTE LEGAL

Se le ofrece la posibilidad de que usted como paciente o como familiar/representante legal del paciente elija voluntariamente la participación del paciente en el proyecto de investigación titulado **"Estudio comparativo, multicéntrico, aleatorio, controlado para evaluar la eficacia de la asistencia ventilatoria ajustada neuralmente (NAVA) frente a la ventilación mecánica protectora convencional en el manejo de pacientes con Insuficiencia Respiratoria Aguda"** que está siendo realizado por el Dr.___________________ del Servicio de ______________________, y que ha sido ya evaluado y aprobado por el Comité Ético de Investigación Clínica del Hospital…………….…………

Antecedentes:

La ventilación mecánica es el tratamiento más importante en el manejo de pacientes que, como su familiar, no pueden mantener por sí mismos la función respiratoria. Debido a una enfermedad que afectaba al pulmón de forma directa o de forma indirecta, fue necesario introducirle un tubo a través de la tráquea para conectarle a un respirador mecánico que le administra aire enriquecido con oxígeno mientras se recupera de su enfermedad. Hay momentos en los que el respirador se encarga completamente de la función respiratoria pero hay otras veces en las que se pueden combinar sus respiraciones con las del respirador para que sus músculos respiratorios se vayan fortaleciendo hasta que su función pulmonar se recupere totalmente.

Tan pronto como el paciente sea capaz de realizar respiraciones efectivas, los médicos que le atienden procederán a iniciar la retirada paulatina del respirador, un proceso que puede durar horas o días. Esta duración depende fundamentalmente de dos factores. El primer factor, y el más importante, es la mejoría de la función pulmonar (que los médicos evalúan regularmente con radiografías y con medidas de la ventilación y de oxigenación de su sangre). El segundo, es la capacidad de adaptación entre la propia respiración del paciente y la respiración artificial que le proporciona el respirador.

En la actualidad, uno de los problemas más frecuentes que tienen los pacientes a la hora de liberarse de la respiración mecánica es la asincronía (o desadaptación) entre sus respiraciones y las del respirador. Las asincronías respiratorias dificultan la transición entre la respiración artificial y la respiración normal, por lo que en muchos casos es necesario interrumpir el proceso de retirada del respirador hasta que el paciente puede adaptarse mejor al respirador. Las asincronías, que pueden ser en algunos casos bastante frecuentes, pueden aumentar la duración de la ventilación artificial, y por tanto el tiempo de hospitalización.

Por el momento, no existe ningún método o técnica de rutina que se haya demostrado que sea mejor para evitar las asincronías y facilitar la retirada del respirador lo antes posible. Sin embargo, hace unos años, se ha introducido en el mercado un nuevo sistema diseñado para facilitar la respiración mecánica. Este sistema, llamado NAVA (por su abreviatura en inglés), es una especie de sonda nasogástrica como la que su familiar tiene colocada a través de su nariz para alimentarle y para evitar que el líquido gástrico pueda entrar en las vías respiratorias. Esta sonda NAVA se conecta por un extremo al respirador. Mediante un sensor situado cerca del extremo colocado en el estómago, registra la actividad muscular del diafragma en cada movimiento respiratorio que el paciente realiza. De esta manera, el respirador reconoce el momento en el que el paciente quiere respirar y hace que la respiración del respirador coincida con la suya, dependiendo siempre del esfuerzo respiratorio que el paciente es capaz de hacer. Hay varios estudios científicos que sugieren que con este sistema, los pacientes ventilados mecánicamente como su familiar toleran bien el respirador y podrían ser liberados antes de la ventilación mecánica.

¿Cuál es el objetivo de este estudio?

En este estudio nos proponemos comprobar y demostrar que el catéter NAVA puede ser la técnica más apropiada para disminuir el número de asincronías y acortar el tiempo que un paciente permanece conectado al respirador.

¿Por qué se le ha pedido que participe?

Se le pide la participación de su familiar en este estudio ya que debido a su enfermedad está ingresado en la Unidad de Cuidados Críticos y conectado a un respirador mecánico, y a partir de las graficas mostradas en su pantalla, hemos detectado que no está bien adaptado al respirador. Con este estudio pretendemos conocer y mejorar la adaptación de los pacientes con insuficiencia respiratoria a los respiradores.

En concreto, esta investigación se está realizando porque hace más de tres años que en España se autorizó el uso de un nuevo sistema de ayuda a la ventilación mecánica llamado NAVA. El NAVA detecta cuando el diafragma quiere empezar y quiere terminar de respirar y se lo dice al respirador el cual te envía aire y oxígeno en el momento más apropiado. Queremos saber si mediante este nuevo método (NAVA) la adaptación (o sincronización) de los pacientes al respirador es mejor que con los métodos tradicionales.
Esta investigación se realizará en varios hospitales de España y lo liderarán médicos expertos nacionales e internacionales en el campo de la insuficiencia respiratoria y la ventilación mecánica del país.

¿En qué consiste su participación? ¿Qué tipo de pruebas o procedimientos se le realizarán?
El principal procedimiento involucrado en este estudio es la ventilación mecánica, la cual ya la tiene porque la necesita por su enfermedad y no por este estudio.
Si cumple los criterios y decide que el paciente participe, será introducido al azar en uno de los dos grupos:
Grupo 1 (control): Ventilación de protección pulmonar convencional
Grupo 2 (estudio): Ventilación mecánica con el método NAVA

Durante todo el tiempo que dure el estudio, para poder comparar posteriormente los dos tipos de ventilación, se registrarán los datos de función respiratoria y hemodinámica por los monitores habitualmente empleados para este fin. Además se realizarán radiografías de tórax y se tomarán muestras de sangre para el control de la función de los pulmones; para la toma de muestras de sangre no será necesario pincharle, ya que se extraerán del catéteres arterial que se coloca habitualmente en estas Unidades. Estas determinaciones coincidirán con las que se realizan habitualmente en estas Unidades para el control del paciente.
Todas estas determinaciones las realizará el médico investigador o la persona del equipo por él designada. Se le solicita permiso para utilizar los datos registrados con fines científicos

Para utilizar el NAVA es necesario colocarle una fina sonda nasogástrica. Esta sonda es idéntica a la sonda nasogástrica que ya tiene colocada, y de hecho le sirve para darle de comer o para aspirar del estómago si lo necesitara y en el caso de que tuviera ya una colocada, se le retiraría al colocarle la del NAVA. La nueva sonda dispone además de unos pequeños electrodos que permiten detectar la actividad eléctrica del diafragma, el principal músculo de la respiración. Estos electrodos van conectados mediante un cable al respirador, el cual detecta inmediatamente cuándo va a iniciar la respiración y le ayuda instantáneamente sin ningún retraso dándole aire y oxígeno que necesita en el momento.
Por último, le informamos que la participación en el presente proyecto no supone ninguna alteración del tratamiento que esté llevando (si lo tiene).

¿Cuáles son los riesgos generales de participar en este estudio?
No se prevé ningún riesgo adicional para su familiar. Dado que se encuentra conectado a un respirador mecánico por motivo de su enfermedad, el estudio no requiere ninguna medida fuera de la rutina habitual. La participación en este no supone ningún riesgo adicional diferente del derivado de la necesidad de ventilación mecánica y del propio proceso de la enfermedad que padece.
Como ya hemos mencionado anteriormente, para utilizar el NAVA es necesario colocarle una sonda nasogástrica. Esta sonda es idéntica a la que ya tiene colocada, y por lo tanto la sonda en si no supone ningún riesgo adicional.

En todo caso el promotor del estudio dispone de una póliza de seguros, nº____________ de la compañía_________________ que se ajusta a la legislación vigente y que le proporcionará la compensación e indemnización en caso de daño a su salud o de lesiones que pudieran producirse en relación con su participación en el estudio.

¿Cuáles son los beneficios de la participación en este estudio?
Basado en conocimientos y observaciones previas parece que la nueva modalidad NAVA permite estar más cómodo con el respirador, necesita menor sedación y se duerme mejor. En el caso de que acceda a participar y se le coloque una sonda NAVA, seguirá siendo ventilado con ayuda de esta sonda mientras necesite el respirador y se encuentre bien adaptado. No podemos garantizar que obtenga beneficios clínicos directos por su participación en el estudio, aunque la colocación de la sonda NAVA no conlleva ningún riesgo añadido a la ventilación mecánica que está recibiendo.

Dado que queremos saber si la ventilación mecánica con ayuda de la sonda NAVA es igual o más eficaz que el manejo tradicional sin NAVA, no le garantizamos que aunque acepte la participación del paciente en el estudio se le coloque esta sonda. Esta decisión será seleccionada por azar, siguiendo instrucciones contenidas en un sobre cerrado del que ninguno de los investigadores tiene conocimiento del contenido hasta después de su apertura. Al final del estudio, la mitad de los participantes habrán sido ventilados de forma convencional y la otra mitad habrán sido ventilados según la información registrada en la sonda NAVA.

¿Qué pasará si decido no participar en este estudio?

La participación del paciente en este estudio es totalmente voluntaria. En caso de que usted decida su no participación en el estudio, esto no modificará el trato y seguimiento que de su enfermedad realicen ni su médico ni el resto del personal sanitario que se ocupa de su enfermedad. Así mismo, usted podrá revocar su consentimiento de participación del paciente en el estudio en cualquier momento, sin tener que dar explicaciones.

¿A quién puedo preguntar en caso de duda?

Es importante que comente con cualquiera de los investigadores de este proyecto los pormenores o dudas que surjan antes de firmar el consentimiento para su participación.

Así mismo, podrá solicitar cualquier explicación que desee sobre cualquier aspecto del estudio y sus implicaciones a lo largo del mismo contactando con el investigador principal del proyecto, el Dr. __________________en el teléfono ____________.

Confidencialidad:

Siguiendo la Ley Orgánica 15/1999, de 13 de Diciembre, de Protección de datos de Carácter Personal, todos sus datos, así como toda la información médica relacionada con su enfermedad será tratada con absoluta confidencialidad por parte del personal encargado de la investigación. Así mismo, si los resultados del estudio fueran susceptibles de publicación en revistas científicas, en ningún momento se proporcionaran datos personales de los pacientes que han colaborado en esta investigación.

Tal y como contempla la Ley de Protección de Datos de Carácter Personal, podrá ejercer su derecho a acceder, rectificar o cancelar sus datos contactando con el investigador principal de este estudio.

El consentimiento informado firmado por el familiar o representante legal
será provisional, y deberá ser confirmado por el paciente en cuanto sea posible.

¿Qué pasará con las muestras biológicas obtenidas durante la investigación?

Durante su participación en este estudio, se le extraerán varias muestras de sangre que se analizarán en ese momento, no procediéndose a su almacenamiento, y que serán tratadas conforme a la Ley 14/2007 de Investigación Biomédica.

CONSENTIMIENTO INFORMADO

Título del Proyecto: **“Estudio comparativo, multicéntrico, aleatorio, controlado para evaluarla eficacia de la asistencia ventilatoria ajustada neuralmente (NAVA) frente a la ventilación mecánica protectora convencional en el manejo de pacientes con Insuficiencia Respiratoria Aguda”**

Investigador principal: ________________________________

Servicio: ____

Don/Doña____

en calidad del familiar, representante legal o allegado del paciente

____

he sido informado por el Dr. ___________________________, colaborador del proyecto de investigación arriba mencionado, y declaro que:

- He leído la Hoja de Información que se me ha entregado
- He podido hacer preguntas sobre el estudio
- He recibido respuestas satisfactorias a mis preguntas
- He recibido suficiente información sobre el estudio

Comprendo que la participación del paciente es voluntaria

Comprendo que el consentimiento informado firmado por el familiar o representante legal será provisional, y deberá ser confirmado por el paciente en cuanto sea posible

Comprendo que todos los datos serán tratados confidencialmente

Comprendo que puedo revocar mi autorización:

- **Cuando quiera**
- **Sin tener que dar explicaciones**
- **Sin que esto repercuta en sus cuidados médicos**

Con esto doy mi conformidad para que participe en este estudio,

Firma del familiar/ representante legal: Firma del Investigador:

Fecha: Fecha

Firma del paciente:
Fecha:
