# Supplementary material for: Neurally adjusted ventilatory assist in patients with acute respiratory failure: study protocol for a randomized controlled trial
Source: Trials. 2016 Oct 13;17:500. doi: 10.1186/s13063-016-1625-5 (PMC5064782; doi:10.1186/s13063-016-1625-5)
Supplement: Additional file 3: — SPIRIT checklist. (PDF 48 kb) [file 13063_2016_1625_MOESM3_ESM.pdf]

SPIRIT

STANDARD PROTOCOL ITEMS: RECOMMENDATIONS FOR INTERVENTIONAL TRIALS

SPIRIT 2013 Checklist: Recommended items to address in a clinical trial protocol and related documents*

Section/item	Item No	Description
**Administrative information**		
Title	1	**Neurally adjusted ventilatory assist in patients with acute respiratory failure: study protocol for a randomized controlled trial**
Trial registration	2a	clinicalTrial.gov (NCT01730794).
Protocol version	3	28 February 2014
Funding	4	Instituto de Salud Carlos III, Madrid, Spain (PI13/0119), CIBER de Enfermedades Respiratorias, Spain (CB06/06/1088), MAQUET-Getinge (Solna, Sweden) and Asociación Científica Pulmón y Ventilación Mecánica (Las Palmas de Gran Canaria, Spain).
Roles and responsibilities	5a	The role and responsibilities of investigators are fully explained in pages 14, 15, 16, and 17 of the manuscript.
	5b	Promotor: Jesús Villar, Hospital Universitario Dr. Negrín, Las Palmas, Spain.
	5c	Study funders have no role in collection, management, analysis, and interpretation of data; writing of the report; and the decision to submit the report for publication.
	5d	See Pages 14 and 15 of the manuscript for composition, roles, and responsibilities of the coordinating centre, steering committee, endpoint adjudication committee, data management team, and other individuals or groups overseeing the trial.
**Introduction**		
Background and rationale	6a	Done. Page 3 of the manuscript.
	6b	Done. Page 3 of the manuscript.
Objectives	7	Specific objectives or hypotheses are stated in Page 3 of the manuscript.
Trial design	8	Description of trial design including type of trial, allocation ratio, and framework is reported in the Abstract (page 2 of the manuscript) and in the Methods section (pages 4 and 5 of the manuscript).

Methods: Participants, interventions, and outcomes

Study setting	9	Description of study settings and country (Spain) are described in Methods section (page 4 and Appendix 1).
Eligibility criteria	10	Inclusion and exclusion criteria for participants are fully reported in page 5 of the manuscript.
Interventions	11a	Interventions for each group with sufficient detail to allow replication, including how and when they will be administered is reported in pages 5 and 6 of the manuscript.
	11b	Criteria for discontinuing or modifying allocated interventions for a given trial participant are stated in the inform consent form.
	11c	Strategies to improve adherence to intervention protocols, and any procedures for monitoring adherence are stated in pages 6 to 9 of the manuscript, and in the Electronic Supplementary Material file.
	11d	There are no specific concomitant care and interventions that are permitted or prohibited during the trial that are applicable to this trial.
Outcomes	12	Primary, secondary, and other outcomes and time point for each outcome are stated in page 10 of the manuscript. Explanation of the clinical relevance of chosen efficacy and harm outcomes is explained in pages 10 and 11 (under the subheading “interim analysis”).
Participant timeline	13	Time schedule of enrolment, interventions, assessments, and follow-up for participants are reported in pages 5 to 10 of the manuscript.<br>A schematic diagram is provided as Figure 1.
Sample size	14	Estimated number of participants needed to achieve study objectives and how it was determined, including clinical and statistical assumptions supporting any sample size calculations is fully reported in page 10 of the manuscript.
Recruitment	15	Strategies for achieving adequate participant enrolment to reach target sample size: not applicable. Patients are consecutively admitted in participating ICUs.

Methods: Assignment of interventions (for controlled trials)

Allocation:

Sequence generation	16a	Method of generating the allocation sequence is stated in page 6 of the manuscript. There is no stratification. To reduce predictability of a random sequence, random assignment is inside individual numbered envelopes that are opaque and sealed. The Project manager has a copy of the random sequence of each center.
Allocation concealment mechanism	16b	As stated above, the mechanism of implementing the allocation sequence is by sequentially numbered, opaque, sealed envelopes provided in blocks of 10 envelopes to each participating center.

Implementation	16c	The allocation sequence was done according to a computer-generated random-number table, as stated in page 6 of the manuscript.
Blinding (masking)	17a	The study is not blinded.

Methods: Data collection, management, and analysis

Data collection methods	18a	A description of the plans for assessment and collection of outcome, and trial data is stated in pages 9 and 11.
	18b	A Project Manager is responsible for promoting patient enrolment and complete follow-up, including list of any outcome data to be collected for participants who discontinue or deviate from intervention protocols.
Data management	19	Plans for data entry, coding, security, and storage, including any related processes to promote data quality is stated in pages 9 and 10 of manuscript.
Statistical methods	20a	Statistical methods for analysing primary and secondary outcomes and other data are clearly stated in page 11 of the manuscript.

Methods: Monitoring

Data monitoring	21a	Details on composition of data monitoring committee, its role, reporting structure and statement of whether it is independent from the sponsor and competing interests are stated in pages 14 and 15 of the manuscript.
	21b	Description of any interim analyses and stopping guidelines is stated in pages 10 and 11 of the manuscript.
Harms	22	Plans for collecting, assessing, reporting, and managing solicited and spontaneously reported adverse events and other unintended effects of trial interventions or trial conduct will be controlled by the Project manager. There is a separate form in the protocol for reporting adverse events (see Electronic supplementary material
Auditing	23	There is no on-site auditing of the trial. Before exporting the data into a computerized data base at the coordinating center, a trained Project Manager will check the completeness and the quality of information. More details in page 11 of the manuscript.

Ethics and dissemination

Research ethics approval	24	The study has been approved by a referral Ethics Committee. According to Spanish legislation, all participating hospitals only require the approval of a coordinating center.
Protocol amendments	25	The Steering Committee and the Project manager will communicate important protocol modifications (when applicable) to relevant parties (investigators, IRBs, trial participants, regulators).
Consent or assent	26a	Participating investigators are responsible for obtaining informed consent from patients or patient’s legal representative (see page 5 of the manuscript.

Confidentiality	27	Patient information is anonymized.
Declaration of interests	28	Competing interest are stated in page 17 of the manuscript.
Access to data	29	As stated in page 17 of the manuscript, the principal investigator (JV), the clinical epidemiologist (LPM) and the Project Manager (RLF) will have access to all the data in the study and take responsibility for the integrity of the data and the accuracy of the data analysis.
Ancillary and post-trial care	30	The trial is covered by an insurance policy, as mandatory by Spanish legislation (Zurich company).
Dissemination policy	31a	Results of the trial will be published in international journals. There is no obligation for communicating the results to individual patients. However, this information will be public.
	31b	All participant investigators have the right to be authors of the final publication.
**Appendices**		
Informed consent materials	32	A copy of the informed consent form (in Spanish) will be provided as additional information at the time of manuscript submission.
Biological specimens	33	Not applicable

*It is strongly recommended that this checklist be read in conjunction with the SPIRIT 2013 Explanation & Elaboration for important clarification on the items. Amendments to the protocol should be tracked and dated. The SPIRIT checklist is copyrighted by the SPIRIT Group under the Creative Commons "Attribution-NonCommercial-NoDerivs 3.0 Unported" license.
