# Supplementary material for: Neurally adjusted ventilatory assist in patients with acute respiratory failure: study protocol for a randomized controlled trial
Source: Trials. 2016 Oct 13;17:500. doi: 10.1186/s13063-016-1625-5 (PMC5064782; doi:10.1186/s13063-016-1625-5)
Supplement: Additional file 4: — Supplementary methods. (DOC 1486 kb) [file 13063_2016_1625_MOESM4_ESM.doc]

***-ELECTRONIC SUPPLEMENTARY METHODS-***

**Neurally adjusted ventilatory assist in patients with acute respiratory failure: study protocol for a randomized controlled trial**

^1,2,3^ **Jesús Villar**, MD, PhD, FCCM jesus.villar54@gmail.com

^4^ **Javier Belda**, MD, PhD fco.javier.belda@uv.es

^1,5^ **Jesús Blanco**, MD, PhD jblancov@saludcastillayleon.es

^1,6^ **Fernando Suarez-Sipmann**, MD, PhD fsuarez.sipmann@surgsci.uu.se

^7^ **José Manuel Añón**, MD, PhD jmaelizalde@gmail.com

^1,8^ **Lina Pérez-Méndez**, MD, PhD linapm1998@yahoo.es

^4^ **Carlos Ferrando**, MD, PhD cafeoranestesia@gmail.com

^9^ **Dácil Parrilla**, MD gudru74@yahoo.es

^9^ **Raquel Montiel**, MD raquelmontiel@gmail.com

^10^ **Ruth Corpas**, MD ruth_corpas@yahoo.es

^7^ **Elena González**, MD, PhD elena03gon@gmail.com

^11^ **David Pestaña**, MD, PhD dpestanalag@hotmail.com

^12^ **Domingo Martínez**, MD dmbct1@gmail.com

^5^ **Lorena Fernández**, MD mlorefer@yahoo.es

^4^ **Marina Soro**, MD, PhD soromarina@gmail.com

^13^ **Miguel Angel García-Bello**, MSc miguelgarciabello@gmail.com

^1,2^ **Rosa Lidia Fernández**, MSc rosalidia.fernandez@gmail.com

^14,15^ **Robert M. Kacmarek**, RTT, PhD rkacmarek@partners.org

**and the NAVa In Acute respiraTORy failure (NAVIATOR) Network***

From

*(1) CIBER de Enfermedades Respiratorias, Instituto de Salud Carlos III, Madrid, Spain;*

*(2) Multidisciplinary Organ Dysfunction Evaluation Research Network, Research Unit, Hospital Universitario Dr. Negrín, Las Palmas de Gran Canaria, Spain;*

*(3) Keenan Research Center for Biomedical Science at the Li Ka Shing Knowledge Institute, St. Michael’s Hospital, Toronto, Canada;*

*(4) Department of Anesthesiology, Hospital Clínico Universitario de Valencia, Valencia, Spain;*

*(5) Intensive Care Unit, Hospital Universitario Río Hortega, Valladolid, Spain;*

*(6) Department of Surgical Sciences, Uppsala University Hospital, Uppsala, Sweden;*

*(7) Intensive Care Unit, Hospital Virgen de La Luz, Cuenca, Spain;*

*(8) Clinical Epidemiology and Biostatistics, Research Unit, Hospital Universitario NS de Candelaria, Santa Cruz de Tenerife, Spain;*

*(9) Intensive Care Unit, Hospital Universitario NS de Candelaria, Santa Cruz de Tenerife, Spain;*

*(10) Intensive Care Unit, Hospital N.S. del Prado, Talavera de la Reina, Toledo, Spain;*

*(11) Department of Anesthesiology, Hospital Universitario Ramón y Cajal, Madrid, Spain;*

*(12) Intensive Care Unit, Hospital Universitario Virgen de la Arrixaca, Murcia, Spain;*

*(13) Biostatistics, Research Unit, Hospital Universitario Dr. Negrín, Las Palmas, Spain;*

*(14) Department of Respiratory Care, Massachusetts General Hospital, Boston, MA, USA;*

*(15) Department of Anesthesiology, Harvard University, Boston, MA, USA.*

*(*) Investigators of the NAVIATOR Network are listed in Appendix 1.*

Address for correspondence: Dr. Jesús Villar

Multidisciplinary Organ Dysfunction Evaluation Research Network

Hospital Universitario Dr. Negrin

Barranco de la Ballena, s/n – 4^th^ floor, South wing

35019 Las Palmas de Gran Canaria

SPAIN

Phone: +(34) 928 449413 Fax: +(34) 928 449813

Email: jesus.villar54@gmail.com

**SUPPLEMENTARY METHODS**

**Guidelines for Positioning the Edi Catheter**

1. First provide reassurance and adequate explanation to the patient.
2. Insert the Edi Module into the SERVO-i and connect the Edi Cable. Perform the Edi Module function check.
3. Measure the distance from the bridge of the Nose (1) to the Earlobe (2) and then to the Xiphoid process (3). This is the NEX measurement. Make a note of it.
4. To confirm the position of the Edi Catheter, open the “Neural access” menu and select “Edi Catheter positioning”.
5. Calculate the insertion distance (Y) for the Edi Catheter. This will depend on whether the Edi Catheter is inserted orally or nasally.

- Insertion distance Y for nasal insertion: 12 Fr 125 cm NEX cm x 0.9 + 18 = Y cm

- Insertion distance Y for oral insertion: 12 Fr 125 cm NEX cm x 0.8 + 18 = Y cm

1. Dip the Edi Catheter into water (no saline) for a few seconds and insert it to the Y distance calculated above. The catheter becomes very slippery after soaking in water. Do NOT use lubricants as this may destroy the Edi Catheter coating and interfere with the measurement of the Edi signal.
2. Connect the Edi Catheter to the Edi Cable. To confirm the position of the Edi Catheter, open the “Neural access” menu and select “Edi Catheter positioning”.
3. Verify the position of the Edi Catheter by analyzing the ECG waveforms. Ideally, P and QRS waves are present in the top leads, while the P waves disappear in the lower leads, where QRS amplitude also decreases. Check that the Edi scale is fixed and that it is set appropriately (≥5 μV).
4. If Edi signals (see Figure E1) are present, observe which leads are highlighted in blue.
5. If the second and third leads are highlighted as shown in Figure E1, secure the Edi Catheter in this position after marking the Edi catheter at its final position and making a note of the distance in cm.
6. If the top leads are highlighted (see Figure E2, left), pull out the Edi Catheter in steps corresponding to the Inter Electrode Distance (IED, measured in millimeters) until the blue highlight appears in the center. Do not exceed four times the IED. Mark the Edi Catheter at its final position. IED length for a 16 Fr catheter is 16 mm.
7. If the bottom leads are highlighted (see Figure E2, right), insert the Edi Catheter further in steps corresponding to the IED until the blue highlight appears in the center. Again, do not exceed four times the IED. Mark the Edi Catheter at its final position.
8. If the Edi signal is very low, there will be no blue highlights. If this happens, evaluate the Edi signal as described in point 15 below.
9. Once the position has been verified, secure the Edi Catheter in position after first checking that the marking on the Edi Catheter is in the right place and observing the ECG waveforms and their blue highlights. Make sure that the Edi Catheter is not secured to the endotracheal tube. Record the insertion length.
10. Evaluate the Edi signal. Note that sedation, hyperventilation, excessively high PEEP and neural disorders can all result in a low or absent Edi signal, even if the Edi Catheter has been perfectly positioned. Let the catheter stay in the determined position until decreases in sedation and/or pressures have recovered an Edi-signal. A maximal Edi should be above 5uV.
11. If possible, perform an expiratory hold and verify that the positive Edi deflection coincides with a negative deflection in the pressure waveform.
12. Edi Catheter positioning should be reconfirmed every 2 to 4 hours. Patient movement, nursing care etc. may change the catheter’s position.
13. The final position of the Edi catheter will be recorded on the data gathering forms.
14. The NAVA catheter should function optimally for 5 days before it needs to be changed.

**Initial NAVA settings**

It is best to initially set NAVA with the head of the bed elevated and the patient in the semirecumbent position. Providing reassurance and adequate explanation to the patient is essential. Since sedation could decrease Edi activity and VT, regardless of NAVA level, sedation should be minimized.

NAVA level will be set initially at zero, then the maximum Edi will be determined as the average level over the next at least 3 to 5 breaths without ventilatory support but with 5 cmH_2_O PEEP. The actual NAVA level will be then titrated by the clinician to achieve the following: 1) an Edi equal to approximately 50% of the maximum Edi, 2) a tidal volume averaging between 4 to 8 ml/kg predicted body weight (PBW), and 3) a respiratory rate averaging between about 15 and 40 per minute. During initial setting of NAVA PaCO_2_ may need to increase to insure appropriate Edi activity. In addition, the trigger sensitivity should be set as sensitive as possible without causing auto-triggering and the maximum pressure limit should be set at 40 cm H_2_O.

**Subsequent Adjustments of NAVA**

As during the initial setting of NAVA the maximum Edi must be determined daily. NAVA level will be set at zero, then the maximum Edi will be determined as the average level over at least the next 3 to 5 breaths without ventilatory support but with 5 cmH_2_O PEEP. The actual NAVA level is then titrated by the clinician to achieve the following: 1) an Edi equal to approximately 50% of the maximum Edi, 2) an average tidal volume of between 4 to 8 ml/kg predicted body weight (PBW), and 3) an average respiratory rate between about 15 and 40 per minute. As with the conventional ventilation arm, patients in the NAVA arm should be assessed daily for meeting criteria for a spontaneous breathing trial.

**Adverse events related to NAVA**

We defined adverse events (AE) as any untoward medical occurrences in a patient during the application of NAVA that are not considered related to the clinical state of the patient.

**Adverse events related to nasogastric (NG) or orogastric (OG) NAVA catheter**

AE related to NG/OG NAVA catheter insertions include:

- the catheter entering the lungs,

- the catheter entering the brain (Serious AE),

- perforation of the esophagus (Serious AE),

- reflux of stomach contents into the esophagus and aspiration.

- Nasal/ pharyngeal bleeding

**Procedures for recording Adverse Events**

All AEs occurring during the study observed by the investigator or reported by the participant or participant’s family, whether or not attributed to study procedures, will be recorded on the data forms. AEs considered related to NAVA as judged by the investigator will be followed until resolution or the event is considered stable. All related AEs that result in a participant’s withdrawal from the study or are present at the end of the study, should be followed up until a satisfactory resolution occurs. At each contact with subjects, the investigator should seek information on AEs by specific questioning and examination. Information on all AEs should be recorded immediately.

If an AE occurs the following information should be provided:

- date and time of onset and end date

- description of the event

- whether study treatment was discontinued

- the reason(s) why the event was classified as serious

- study centre

- who identified the event

- subject name

- investigator assessment of relatedness to study procedures or to disease

- severity

- the type and duration of follow up for subjects after AE

**Reporting procedures for serious adverse events**

- All AE reported will be sent to the DSMB as well as the IRB for review.
- All serious events will be sent within 24 hours after being received to the coordinating center and IRB.
- Non-serious events will be sent within one week of reception to the coordinating center and IRB.
- All unexpected and related or possibly related adverse events will be reported to the DSMB and IRB

**TABLE E1. The Riker Sedation Agitation Scale (SAS).** *NOTE: Prior to conducting a Spontaneous Breathing Trial, the SAS scale should be a score of 3 to 5, ideally 4*

| ***Score*** | ***Level of sedation/agitation*** | ***Response*** |
| --- | --- | --- |
| 7 | Dangerous agitation | Pulling endotracheal tube, climbing out of bed, etc. |
| 6 | Very agitated | Does not calm, requires restraints |
| 5 | Agitated | Calms to instructions |
| 4 | Calm and cooperative | Obeys commands |
| 3 | Sedated | Difficult to verbally arouse |
| 2 | Very sedated | Arouses to physical stimuli |
| 1 | Unarousable | Minimal or no response to noxious stimuli |

**TABLE E2. The Ramsay Sedation Scale. *NOTE: Prior to conducting a Spontaneous Breathing Trial, the Ramsey Sedation Scale should be a score of 2 to 4, ideally 3.***

| ***Clinical Score*** | ***Patient Characteristics*** |
| --- | --- |
| 1 | Anxious, agitated, or restless |
| 2 | Cooperative, oriented, and tranquil |
| 3 | Sedated but responds to commands |
| 4 | Asleep; brisk response to light tap or loud auditory stimulus |
| 5 | Asleep; sluggish response to light tap or loud auditory stimulus |
| 6 | Asleep; no response to painful stimuli |

**TABLE E3. The Richmond Agitation Sedation Scale (RASS). Prior to conducting a Spontaneous Breathing Trial, the RASS should be between –2 to +1, ideally 0.**

| **Score** | **Term** | **Description** |  |
| --- | --- | --- | --- |
| +4 | Combative | Overtly combative, violent, immediate danger to staff |  |
| +3 | Very agitated | Pulls or removes tube(s) or catheter(s); aggressive |  |
| +2 | Agitated | Frequent non-purposeful movement, fights ventilator |  |
| +1 | Restless | Anxious but movements not aggressive vigorous |  |
| 0 | Alert and calm |  |  |
| -1 | Drowsy | Not fully alert, but has sustained awakening  (eye-opening/eye contact) to *voice* (>10 s) | Verbal Stimulation |
| -2 | Light sedation | Briefly awakens with eye contact to *voice* (<10 s) | Verbal Stimulation |
| -3 | Moderate sedation | Movement or eye opening to *voice* (but no eye contact) | Verbal Stimulation |
| -4 | Deep sedation | No response to voice, but movement or eye opening  to *physical* stimulation | Physical Stimulation |
| -5 | Unarousable | No response to *voice or physical* stimulation | Physical Stimulation |

# **Procedure for RASS Assessment**

- Observe patient
  - Patient is alert, restless, or agitated………………………………………score 0 to +4
- If not alert, state patient’s name and *say* to open eyes and look at speaker:
  - Patient awakens with sustained eye opening and eye contact………………score –1
  - Patient awakens with eye opening and eye contact, but not sustained……...score –2
  - Patient has any movement in response to voice but no eye contact…………score –3
- When no response to verbal stimulation, physically stimulate patient by shaking shoulder and/or rubbing sternum:
  - Patient has any movement to physical stimulation………………………… score –4
  - Patient has no response to any stimulation…………………………..........…score –5

**FIGURE E1. Edi catheter position**

**FIGURE E2.** *Left picture*: top leads are highlighted. Edi catheter should be pulled out. *Right picture*: bottom leads are highlighted. Edi catheter should be inserted further.
